# AAV-mediated rescue of *Eps8* expression *in vivo* restores hair-cell function in a mouse model of recessive deafness

**DOI:** 10.1016/j.omtm.2022.07.012

**Published:** 2022-07-31

**Authors:** Jing-Yi Jeng, Adam J. Carlton, Richard J. Goodyear, Colbie Chinowsky, Federico Ceriani, Stuart L. Johnson, Tsung-Chang Sung, Yelena Dayn, Guy P. Richardson, Michael R. Bowl, Steve D.M. Brown, Uri Manor, Walter Marcotti

**Affiliations:** 1 School of Bioscience, University of Sheffield, Sheffield S10 2TN, UK; 2 Sussex Neuroscience, School of Life Sciences, University of Sussex, Falmer, Brighton BN1 9QG, UK; 3 Waitt Advanced Biophotonics Center, Salk Institute for Biological Studies, 10010 N. Torrey Pines Road, La Jolla, CA 92037, USA; 4 Transgenic Core, Salk Institute for Biological Studies, 10010 N. Torrey Pines Road, La Jolla, CA 92037, USA; 5 Mammalian Genetics Unit, MRC Harwell Institute, Harwell Campus, Oxfordshire OX11 0RD UK; 6 Neuroscience Institute, University of Sheffield, Sheffield S10 2TN, UK

**Keywords:** mouse, auditory, cochlea, deafness, AAVs, gene therapy, stereocilia, exocytosis, Eps8, development

## Abstract

The transduction of acoustic information by hair cells depends upon mechanosensitive stereociliary bundles that project from their apical surface. Mutations or absence of the stereociliary protein EPS8 cause deafness in humans and mice, respectively. *Eps8* knockout mice (*Eps8*^*−/−*^) have hair cells with immature stereocilia and fail to become sensory receptors. Here, we show that exogenous delivery of *Eps8* using Anc80L65 in P1–P2 *Eps8*^*−/−*^ mice *in vivo* rescued the hair bundle structure of apical-coil hair cells. Rescued hair bundles correctly localize EPS8, WHIRLIN, MYO15, and BAIAP2L2, and generate normal mechanoelectrical transducer currents. Inner hair cells with normal-looking stereocilia re-expressed adult-like basolateral ion channels (BK and KCNQ4) and have normal exocytosis. The number of hair cells undergoing full recovery was not sufficient to rescue hearing in *Eps8*^*−/−*^ mice. Adeno-associated virus (AAV)-transduction of P3 apical-coil and P1–P2 basal-coil hair cells does not rescue hair cells, nor does Anc80L65-Eps8 delivery in adult *Eps8*^*−/−*^ mice. We propose that AAV-induced gene-base therapy is an efficient strategy to recover the complex hair-cell defects in *Eps8*^*−/−*^ mice. However, this therapeutic approach may need to be performed *in utero* since, at postnatal ages, *Eps8*^*−/−*^ hair cells appear to have matured or accumulated damage beyond the point of repair.

## Graphical abstract

**Figure:**
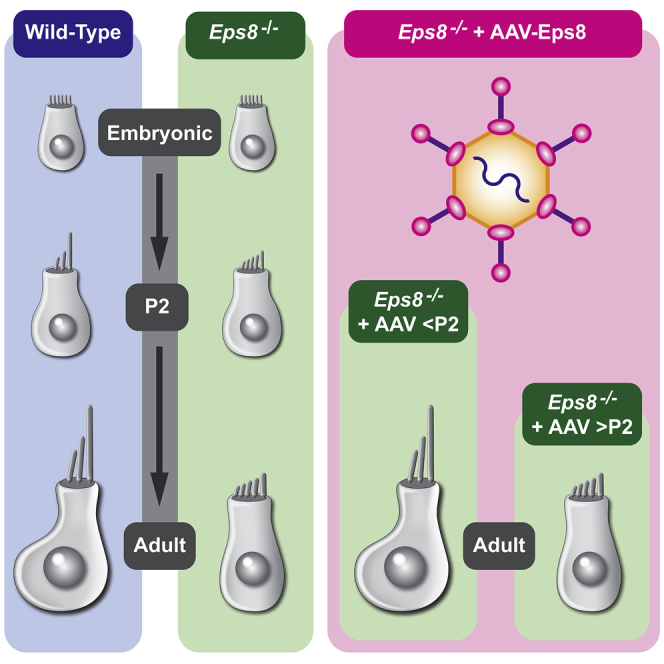

## Teaser

The actin-binding protein EPS8 is key for the elongation of the hair-cell stereociliary bundles, which transduce sound into electrical signals. Loss of EPS8 leads to short stereocilia, abnormal hair-cell function, and deafness. We show that the adeno-associated virus (AAV) delivery of exogenous *Eps8* can rescue hair-cell function.

## Introduction

Hearing loss (HL) is a very common condition that can arise as a congenital birth defect or can develop postnatally. According to a recent report from the World Health Organization (WHO), by 2050 it is projected that nearly 2.5 billion people globally will suffer some degree of HL, with genetic predisposition being a key factor. Indeed, in developed countries, pre-lingual HL has an incidence of ∼1 in 500, and in ∼80% of cases there is a genetic cause.^1^ To date, mutations in 124 genes have been identified as causing monogenic non-syndromic HL in humans, several of which are expressed in the stereociliary bundles of hair cells.^2^^,^^3^

Cells use actin-based plasma membrane protrusions to detect cues from their environment, which are then used to regulate several physiological processes such as motility, adhesion, and even mechanosensation.^4^, ^5^, ^6^ In the cochlea, specialized microvilli-like structures emerge from the apical surface of the sensory inner hair cells (IHCs) and outer hair cells (OHCs). These membrane protrusions, called stereocilia, are key for transducing acoustic information into the hair-cell receptor potentials, which are then used to drive the action potential activity in the auditory fibers.^7^ This mechanoelectrical transduction is operated by mechanically gated ion channels positioned near the tips of hair-cell stereocilia.^8^ Stereocilia are composed of a compact array of parallel and uniformly polarized actin filaments,^5^^,^^9^^,^^10^ and the length of each stereocilium is scaled precisely to give rows of stereocilia (hair bundle) that form a staircase-like architecture. The hair-cell stereociliary bundles are composed of three rows of stereocilia, which are cross-linked by several types of extracellular links.^11^^,^^12^ The architecture of the hair bundles is so precise that the height and width of stereocilia within each row is similar, not only within a single hair bundle but also between the bundles of adjacent hair cells. In altricial rodents, this sophisticated control over the growth of stereocilia occurs during late embryonic and early postnatal stages,^13^, ^14^, ^15^ and is tightly regulated by several actin-binding proteins and unconventional myosin motors.^5^^,^^12^ One of the key molecules regulating the length of the actin filaments in the stereocilia of hair cells is epidermal growth factor receptor substrate 8 (EPS8).^16^, ^17^, ^18^

EPS8 is an actin-regulatory protein endowed with actin bundling and capping activities.^19^^,^^20^ In cochlear hair cells, EPS8 is primarily found at the tips of the longest stereocilia^18^ as part of a protein complex formed by MYO15A, WHIRLIN, GPSM2, and GNAI3.^16^^,^^17^^,^^21^, ^22^, ^23^, ^24^ EPS8 is also key, together with MYO15A, to localize BAIAP2L2 at the tip of the shorter rows of stereocilia, which is required for the maintenance of the hair bundles.^25^ In the absence of EPS8, mice are deaf since several key morphological and physiological characteristics of the hair cells are halted at very immature stages of development.^17^ In line with these observations in mice, humans with mutations in *Eps8* are profoundly deaf.^26^^,^^27^

Here, we investigated the ability of adeno-associated virus (AAV) vector Anc80L65^28^ to rescue the complex morphological and functional defects present in hair cells from *Eps8*^*−/−*^ mice. This AAV vector, containing the coding sequence of wild-type *Eps8* with an N-terminal FLAG tag (referred to hereafter as Anc80L65-Eps8), was injected through the round window membrane into the cochlea of P1–P2 mice. We found that transduced hair cells expressing exogenous EPS8 showed a variable degree of hair bundle morphological recovery. Transduced hair cells with fully rescued hair bundles had normal mechanoelectrical transduction and were able to progress in their maturation. We propose that AAV delivery of exogenous *Eps8 in vivo* is an efficient method to recover the complex hair-cell dysfunctions in *Eps8*^*−/−*^ mice, but it may need to be performed *in utero* to recover hearing function, as the work presented here suggests the existence of a critical window for functional hair-cell recovery.

## Results

### Transduction of cochlear hair cells using AAVs *in vivo*

**Figure 1:**
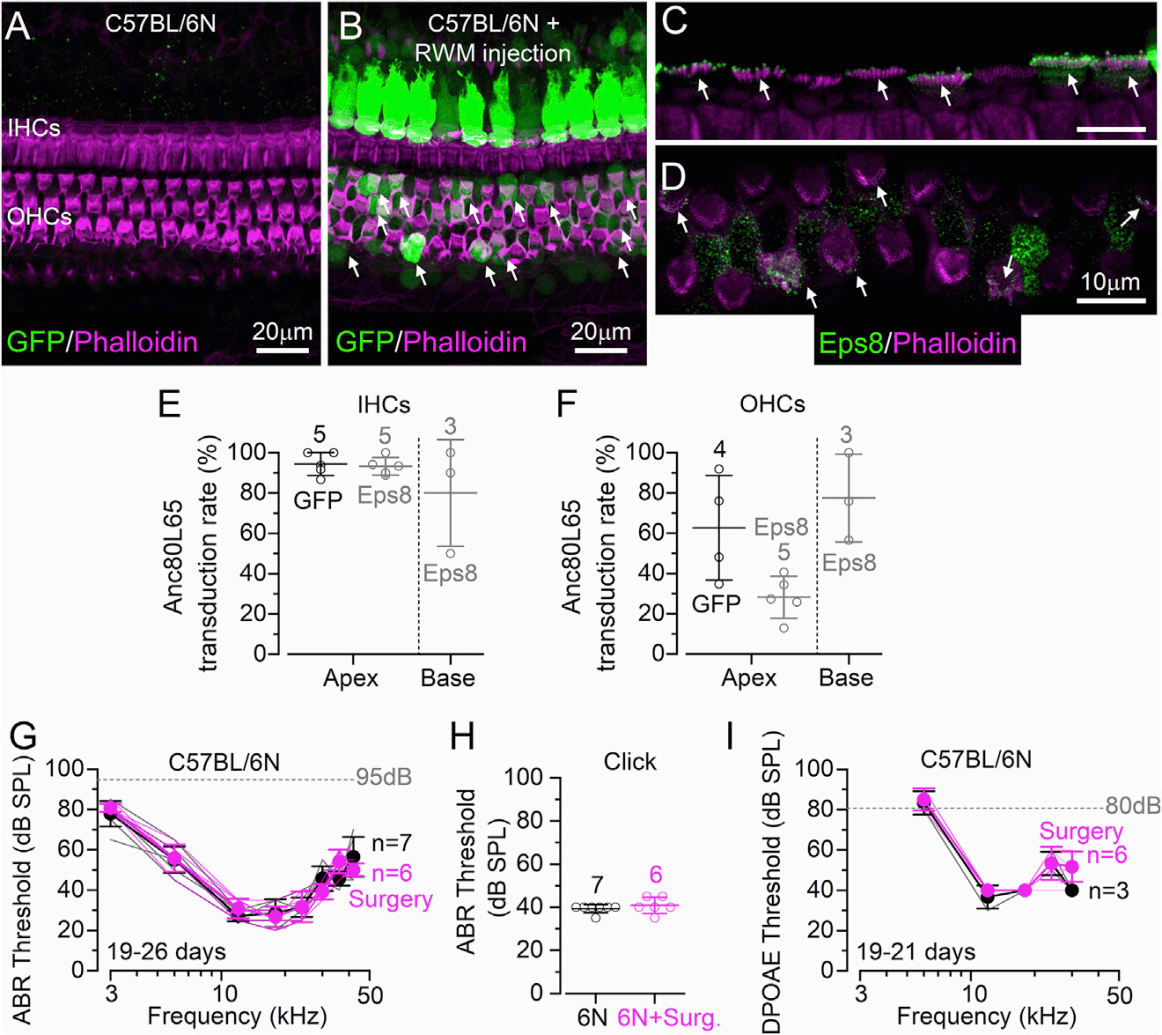
Transduction efficiency of Anc80L65 in cochlear hair cells (A and B) Confocal images obtained from the apical coil of the cochlea (9- to 12-kHz region) from wild-type mice (C57BL/6N strain), which were either not injected (A) or injected (B) *in vivo* with Anc80L65-GFP (2.58 × 10^13^ vg/mL) through the round window membrane (RWM). Cochleae, which were dissected from the above mice at P21, were fixed, stained with Texas red phalloidin, and imaged for GFP. While all IHCs were GFP positive, only some of the OHCs expressed GFP (some of them indicated by an arrow). Scale bar represents 20 μm and applies to both panels. (C and D) Confocal images obtained as described in (A) and (B) but using *Esp8*^*−/−*^ mice injected with Anc80L65-Eps8 (8.90 × 10^12^ vg/mL). Esp8 primarily localizes at the tip of the taller stereocilia of most of the IHCs and some OHCs (indicated by arrows). (E and F) Viral-transduction rates in apical and basal IHCs (E) and OHCs (F) determined from the number of GFP-positive hair cells (injected Anc80L65-GFP, as in B) and Eps8-positive hair cells (injected Anc80L65-Eps8, as in C and D) and in each cochlear preparation normalized by the total hair cells identified with phalloidin. In (E) and (F), the number of mice/cochleae (one mouse = one cochlea) is indicated next to the data. (G) ABR thresholds for frequency-specific pure tone stimulation from 3 to 42 kHz recorded from non-injected (black) and injected (magenta: surgery and RWM injection of Anc80L65-GFP) C57BL/6N wild-type mice at 19–26 days. The number of mice tested is shown. The dashed line represents the upper threshold limit of our system, 95 dB. Single animal recordings are plotted as faded lines. (H) Average ABR thresholds for click stimuli recorded from the same mice described in (G). Number of mice tested is shown above the average data, and single data points are plotted as small open symbols. (I) DPOAE thresholds measured from a subset of mice used in (E). The frequency range tested was between 6 and 24 kHz. Dashed line: upper threshold limit of our system, 80 dB. Single-animal recordings are shown as faded lines. Data are mean ± SD.

The transduction efficiency of the synthetic adeno-associated viral vector Anc80L65 was determined from the number of GFP-positive hair cells (injected Anc80L65-GFP, five mice) and Eps8-positive hair cells (injected Anc80L65-Eps8, five mice) into the perilymphatic space via the round window membrane (RWM) of P1–P2 C57BL/6N (Figures 1A and 1B) and *Eps8*^*−/−*^ (Figures 1C and 1D) mice. Transduced hair cells were identified by the expression of GFP in their cell body (Figure 1B) or the immuno-localization of EPS8 at their stereociliary bundles (Figures 1C and 1D) using confocal microscopy. In the apical coil of the cochlea, which was used for all the *ex vivo* measurements, viral-transduction rate for the combined Anc80L65-GFP and Anc80L65-Eps8 was ∼94% in IHCs, but only ∼44% in OHCs (p < 0.0001, t test; Figures 1E and 1F). In the basal cochlear coil, transduction was comparable between the two hair-cell types (≥50%). To investigate whether the surgery procedure and the RWM injections of Anc80L65-GFP caused any hearing deficits, we measured the auditory brainstem responses (ABRs) from injected and non-injected C57BL/6N wild-type mice between P19 and P26 (Figures 1G–1I). ABRs, which measure the summed sound-induced activity of the auditory pathway,^29^ showed thresholds for tone and click stimuli that were indistinguishable between injected and non-injected mice (Figure 1G, p = 0.8852, two-way ANOVA; Figure 1H, p = 0.3574, t-test). Distortion product otoacoustic emissions (DPOAEs), which are a readout of active non-linear cochlear amplification due to OHC activity,^30^ were also comparable between the two experimental conditions (Figure 1I, p = 0.0527, two-way ANOVA). Because transduction efficiency of Anc80L65 was higher in apical-coil IHCs, most of the following experiments were performed in this region.

### Anc80L65-EPS8 promotes stereocilia growth in *Eps8* knockout mice

**Figure 2:**
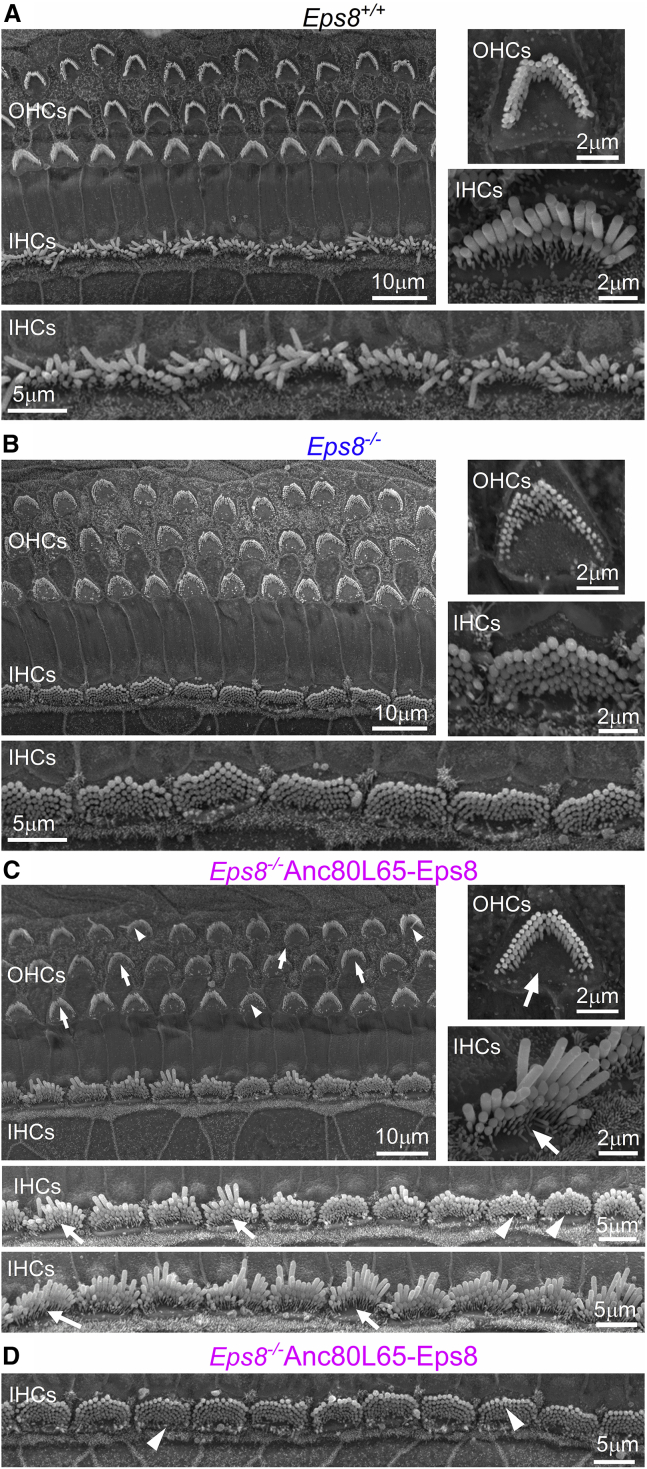
Hair bundle morphology in hair cells from transduced *Eps8*^*−/−*^ mice with Anc80L65-Eps8 (A–C) Scanning electron micrographs showing typical examples of the hair bundle structure of apical-coil OHCs and IHCs in adult wild-type mice (*Eps8*^*+/+*^, P24: A), *Eps8*^*−/−*^ mice (*Eps8*^*−/−*^, P24: B), and P22 *Eps8*^*−/−*^ mice that were transduced with Anc80L65-Eps8 *in vivo* (C) at P1–P2. Note that, generally, mouse hair bundles are composed of three rows of stereocilia: tall, intermediate, and short (A, see insets). In *Eps8*^*−/−*^ mice, both IHCs and OHCs have extra rows of short stereocilia (B, see insets), which is in agreement with previous findings.^17^ In (C), arrows indicate hair bundles from *Eps8*^*−/−*^ mice that have regained the normal morphology. Arrowheads indicate hair bundles that show a variable degree of recovery. (D) Images from a P22 *Eps8*^*−/−*^ mouse that was transduced with Anc80L65-Eps8 *in vivo* at P3. Almost every IHC retained an immature hair bundle morphology (arrowheads), with some stereocilia having elongated (arrows).

EPS8 has been shown to localize at the tips of stereocilia of both IHCs and OHCs and is essential for their normal elongation. In adult wild-type mice, both IHCs and OHCs are composed of three rows of stereocilia (tall, intermediate, and short), which can be easily identified using scanning electron microscopy (Figure 2A; see also Fettiplace and Kim^7^). In *Eps8*^*−/−*^ adult mice, the hair bundles of both IHCs and OHCs are comparable with those present during the early stages of immature development, displaying several rows of very short stereocilia (Figure 2B). Therefore, we investigated whether the injection of Anc80L65 containing the coding sequence of wild-type Eps8 with an N-terminal FLAG tag (referred to hereafter as Anc80L65-Eps8) in the RWM in P1–P2 pups was able to rescue the normal growth of stereocilia in *Eps8*^*−/−*^ mice. We found that about 3 weeks after AAV delivery in *Eps8*^*−/−*^ mice (i.e., at P22–P26), the hair bundles of both IHCs and OHCs showed largely improved structure (Figure 2C). However, the degree of recovery was quite variable, with some hair bundles from both IHCs (Figures 2C and S1) and OHCs (Figures 2C and S2) being indistinguishable from those present in wild-type mice (Figure 2A), and others showing an intermediate or a very immature phenotype (Figures 2C, S1, and S2). When mice were injected with Anc80L65-Eps8 at P3, we observed almost no recovery (Figure 2D).

**Figure 3:**
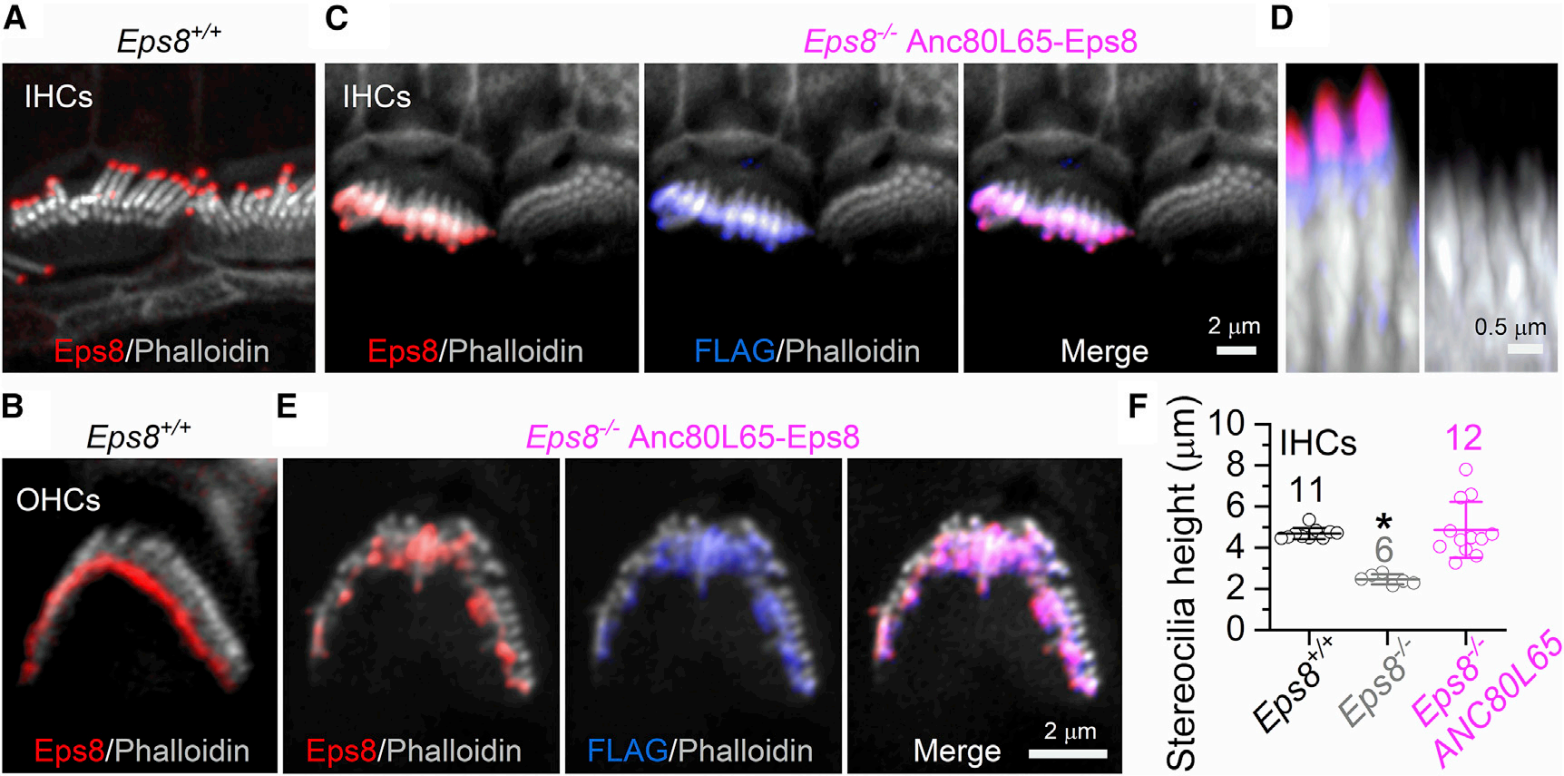
EPS8 localizes to stereocilia tips of AAV-transduced *Eps8*^*−/−*^ mice (A–E) Confocal images of the hair bundles from apical-coil IHCs (A, C, D) and OHCs (B and E) of P18 *Eps8*^*+/+*^ mice (A and B) and P21 *Eps8*^*−/−*^ mice that underwent injection with Anc80L65-EPS8 at P2 (C-E). Hair cells were immunostained for EPS8 and, for the Anc80L65-EPS8 transduced mice, also for FLAG tag. Note the characteristic localization of Eps8 at the tip of the taller row of stereocilia. For mice transduced with Anc80L65-EPS8 (C and D), one transduced IHC is shown next to an IHC that is not expressing the exogenous EPS8. The FLAG tag, which is fused to the N terminus of the viral vector-expressed protein, was also localized at the taller stereocilia tips (C and E, middle panels) as also shown in the merge images (C and E, right panels). The elongation of the stereocilia in *Eps8*^*−/−*^ mice that underwent transduction is better highlighted in the orthogonal projections images of the stereocilia (D). (F) Height of the tallest row of stereocilia, which is that expressing EPS8 in adult mice, in IHCs from the different experimental conditions: *Eps8*^*+/+*^ (four cochleae), *Eps8*^*−/−*^ (four cochleae), and following the injection of Anc80L65-EPS8 (*Eps8*^*−/−*^ + Anc80L65) (seven cochleae). Number of mice/cochlea tested (one mouse = one cochlea) is shown above the average data and single data points are plotted as small open symbols. ANOVA Tukey’s post test: p = 0.0003 and p < 0.0001 for *Eps8*^*−/−*^ compared with *Eps8*^*+/+*^ and *Eps8*^*−/−*^ + Anc80L65, respectively; p= 0.8863 for *Eps8*^*+/+*^ compared with *Eps8*^*−/−*^ + Anc80L65. Data are means ± SD.

We then established whether the rescued hair bundles from *Eps8*^*−/−*^ mice transduced with Anc80L65-Eps8 at P1–P2 showed a normal distribution of EPS8 using immunofluorescence labeling with antibodies against EPS8 and, for the transduced hair cells, the FLAG tag fused to the N terminus of EPS8 (see section “materials and methods”). Similar to wild-type mice (Figures 3A and 3B; see also Zampini et al.^17^ and Furness et al.^18^), hair cells from *Eps8*^*−/−*^ mice transduced with Anc80L65-Eps8 were able to localize EPS8 at the tips of their taller stereocilia (Figures 3C, 3D, and3E). Transduced hair cells also showed a stereocilia tip localization of the FLAG tag, which overlapped with the EPS8 staining (Figures 3C, 3D, and 3E). The length of the tallest row of stereocilia in the IHCs was quantified, as these were easier to measure than those of OHCs, by labeling the actin core with phalloidin (see section “materials and methods”). We found that the stereocilia height in IHCs from non-rescued *Eps8*^*−/−*^ mice was significantly smaller than that measured in cells from wild-type and *Eps8*^*−/−*^ mice with rescued hair bundles (p < 0.0001, one-way ANOVA; Figure 3F). The height of the bundles of wild-type and *Eps8*^*−/−*^ mice with fully rescued hair bundles were not significantly different (p = 0.8863).

**Figure 4:**
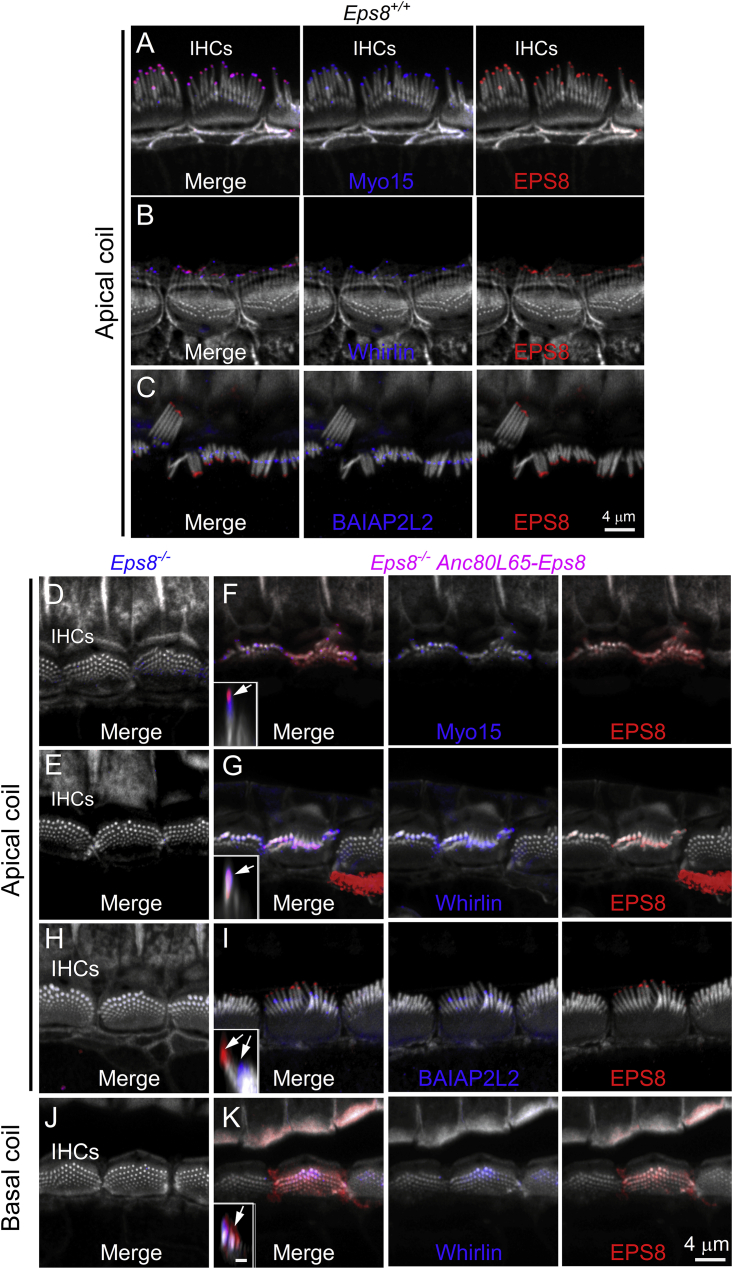
Hair bundle proteins involved in stereociliary elongation (A–C) Confocal images of the hair bundles from apical-coil IHCs of P25–P29 control *Eps8*^*+/+*^ mice. Adult cochleae were immunostained for EPS8 (red) together with MYO15 (A), WHIRLIN (B), and BAIAP2L2 (C) (blue). All proteins showed the characteristic localization at the tip of the stereocilia, which were labeled with phalloidin (white). (D–K) Confocal images of the hair bundles from apical-coil IHCs of P25-P29 *Eps8*^*−/−*^ mice (D, E, H) and *Eps8*^*−/−*^ mice transduced with Anc80L65-EPS8 at P1–P2 (F, G, I). For *Eps8*^*−/−*^ mice, the individual color channels used in the “Merge” images in (D), (E), (H), and (J) are shown in Figure S6. In rescued hair bundles, both MYO15 and WHIRLIN localize at the tips of the taller stereocilia. Also, BAIAP2L2 exhibits its normal distribution at the top of the shorter transducing stereocilia. (J and K) Confocal images as shown in the above panels but from basal-coil IHCs of the adult cochlea of *Eps8*^*−/−*^ mice transduced with Anc80L65-EPS8 at P1. Note that the hair bundle of the transduced IHC retained an immature morphology, and that both EPS8 and WHIRLIN are localized throughout the several rows of the short stereocilia. Scale bar in the inset of (K), which also applies for panels (F), (G), and (I), represents 1 μm.

EPS8, MYO15, and WHIRLIN are all hair bundle proteins that localize at the tip of the taller rows of stereocilia of P25–P29 wild-type mice (Figures 4A–4C), and are involved in their selective lengthening and widening relative to the other shorter rows by promoting F-actin polymerization at their tips.^16^^,^^31^^,^^32^ In apical-coil IHCs, we found that both MYO15 and WHIRLIN were absent in P25–P29 *Eps8*^*−/−*^ mice at the tip of the tallest stereocilia (Figures 4D and 4E), but co-localized with EPS8 at the tips of the taller stereocilia in P25–P29 *Eps8*^*−/−*^ mice transduced with Anc80L65-Eps8 at P1 (Figures 4F and 4G). Although the growth and maintenance of the transducing shortest two rows of stereocilia is less well understood, they require EPS8 during early stages of development^17^^,^^33^ and several other proteins, including the I-BAR protein BAIAP2L2,^25^ EPS8L2,^18^ and MYO15.^33^^,^^34^ We found that only the rescued hair bundles from P25–P29 *Eps8*^*−/−*^ mice were able to localize BAIAP2L2 in their shorter rows of stereocilia (Figures 4F and 4I), which has been shown to be dependent not only on EPS8 but also on the motor protein MYO15.^25^

Cellular differentiation, which is defined by Atoh1 expression during embryonic stages, changes along the length of the cochlea with basal hair cells being about 2–3 days ahead compared with apical cells.^35^ Because of this differential development along the cochlea, at P0–P1 basal-coil, but not apical-coil, hair cells exhibit a functional mechanoelectrical transducer current and larger basolateral membrane currents.^36^, ^37^, ^38^ We found that, in contrast to apical-coil IHCs, the bundles of hair cells located toward the basal end of the cochlea of *Eps8*^*−/−*^ mice transduced with Anc80L65-Eps8 did not elongate, and both EPS8 and WHIRLIN showed a more diffuse distribution along the length of several rows of stereocilia (Figures 4J and 4K).

Despite the morphological recovery of the hair bundles of at least some of the hair cells from *Eps8*^*−/−*^ mice transduced with Anc80L65-Eps8 at P1–P2, we did not observe any functional recovery of hearing measured using either ABR or DPOAE (Figure S3).

### Anc80L65-Eps8 rescues the mechanotransducer currents in *Eps8*^*−/−*^ mice

Hair cells from *Eps8*^*−/−*^ mice have been shown to have a wide range of morphological and physiological dysfunctions.^17^ Therefore, we investigated whether the absence of hearing in *Eps8*^−/−^ mice injected with Anc80L65-Eps8 at P1–P2 was due to the limited ability to restore key functional characteristics of developing hair cells separate from hair bundle structure. To test this possibility, we focused the following work on the IHCs since they show a higher viral-transduction rate than OHCs (Figure 1), and their taller stereocilia makes it easier to recognize their morphology using our upright microscope with differential interference contrast (DIC) optics and a water immersion objective (see section “materials and methods”). In the *Eps8*^*−/−*^ mice transduced with Eps8, the following experiments were performed from IHCs that exhibited three well-defined rows of stereocilia with homogeneous height, which are about 21% of those viewed under the field of view of our microscopes.

**Figure 5:**
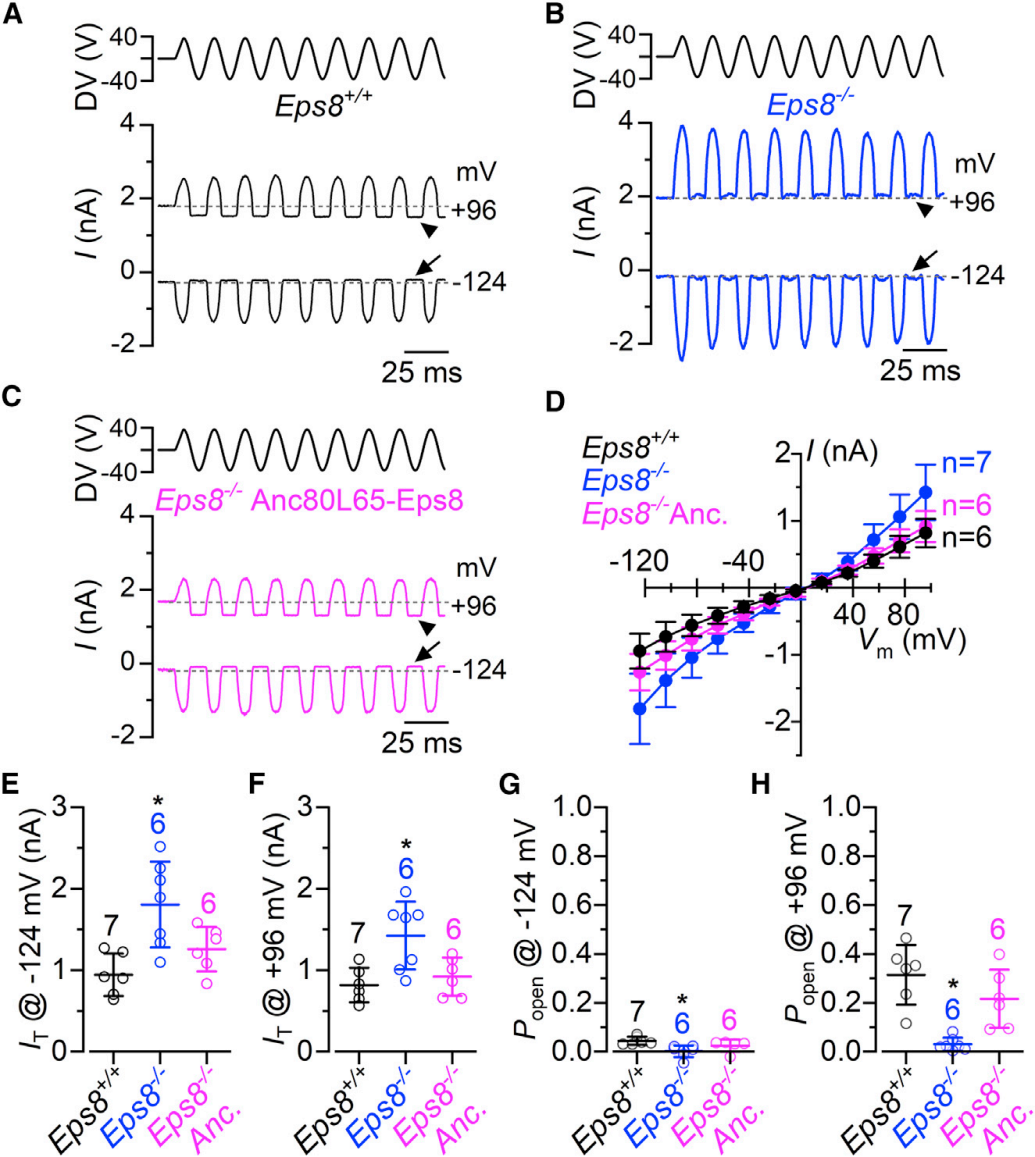
Anc80L65-Eps8 rescue of mechanoelectrical transduction in *Eps8*^*−/−*^ mice (A–C) Saturating MET currents in apical P9 IHCs from control *Eps8*^*+/+*^ (A), *Eps8*^*−/−*^ (B), and *Eps8*^*−/−*^ injected with Anc80L65-Eps8 (C) mice in response to 50-Hz sinusoidal force stimuli to the hair bundles at membrane potentials of −124 and +96 mV. Driver voltage (DV) stimuli to the fluid jet are shown above the traces, with positive deflections of the DV being excitatory. The arrows and arrowheads indicate the closure of the transducer channel in response to inhibitory bundle stimuli at −124 and +96 mV, respectively. (D) Peak-to-peak MET current-voltage curves obtained from P9 IHCs of the mouse models described in (A)–(C). Recordings were obtained by mechanically stimulating the hair bundles of IHCs while stepping their membrane potential from −124 to +96 mV in 20-mV increments. (E and F) Maximal size of the MET current measured at −124 mV (E) and +96 mV (F) from *Eps8*^*+/+*^, *Eps8*^*−/−*^, and *Eps8*^*−/−*^ injected with Anc80L65-Eps8. One-way ANOVA: p = 0.0031 (E) and p = 0.0057 (F) is indicated by the star symbol. For post test, see section “results”. (G and H) Resting open probability (*P*_o_) of the MET current in IHCs from the three above mouse models at the holding of −124 mV (G) and +96 mV (H). The resting current is given by the holding current minus the current present during inhibitory bundle deflection. One-way ANOVA: p = 0.0002 (G) and p = 0.0138 (H) is indicated by the star symbol. For post test, see section “results”. Data are plotted as means ± SD.

The functional rescue of the hair bundles from *Eps8*^*−/−*^ mice transduced with Anc80L65-Eps8 at P1–P2 was investigated by recording the mechanoelectrical transducer (MET) current. MET currents were recorded from P9 apical-coil IHCs by displacing their stereociliary bundles using a 50-Hz sinusoidal force stimulus from a piezo-driven fluid jet.^39^^,^^40^ Moving the bundles toward the taller stereocilia (i.e., in the excitatory direction) elicited a large inward MET current in IHCs from wild-type mice (Figure 5A), *Eps8*^*−/−*^ mice (Figure 5B), and *Eps8*^*−/−*^ mice transduced with the viral vector (Figure 5C). By stepping the membrane potential from −124 mV to more depolarized values in 20-mV increments, the transducer current decreased in size at first and then reversed near 0 mV in all experimental conditions (Figure 5D), consistent with the non-selective permeability of MET channels to cations. At −124 mV, the maximal MET current in IHCs from wild-type mice (−944 ± 260 pA, n = 6 at −124 mV) was significantly smaller than that measured in *Eps8*^*−/−*^ mice (−1,807 ± 525 pA, n = 7, p = 0.0026, one-way ANOVA, Tukey’s post test; Figure 5E). The larger MET current in *Eps8*^*−/−*^ mice correlates with their larger number of stereociliary rows, and most likely MET channels, as previously shown.^17^ Hair cells from AAV-transduced *Eps8*^*−/−*^ mice showed an MET current that was no longer significantly higher than wild-type mice (−1,259 ± 273 pA, n = 6, p = 0.3546, Tukey’s post test; Figure 5E). The maximal MET current at +96 mV (Figure 5F) follows a similar trend to −124 mV. *Eps8*^*−/−*^ mice transduced with Anc80L65-Eps8 showed a similar current size at +96 mV (+921 ± 234 pA, n = 6) to wild-type (+818 ± 212 pA, n = 6, p = 0.8339) but was significantly reduced compared with that measured in cells from *Eps8*^*−/−*^ mice (+1,424 ± 416 pA, n = 7, p = 0.0076).

One important feature of the MET channel is its resting open probability (*P*_o_), which allows the flow of an MET current through open transducer channels in the absence of mechanical stimulation. The resting MET current can be measured as the difference between the holding current and the current present during inhibitory bundle deflection, which completely closes the MET channel (Figures 5A–5C). At both negative and positive membrane potentials, the resting MET current was evident in IHCs from wild-type mice (Figures 5A, 5G, and 5H), but absent in cells from *Eps8*^*−/−*^ mice (Figures 5B, 5G, and 5H), in agreement with a previous study.^17^ The larger resting MET current at positive membrane potentials is due to an increased *P*_o_ of the MET channel resulting from a reduced driving force for Ca^2+^ influx.^39^^,^^41^ *Eps8*^*−/−*^ mice transduced with Anc80L65-Eps8 showed a rescue of the resting MET current, which was not significantly different from that measured in IHCs of wild-type mice (p = 0.5610, at −124 mV, p = 0.2150; at +96 mV, one-way ANOVA, Tukey’s post test; Figures 5C, 5G, and 5H).

The above data indicate that injection of Anc80L65-Eps8 into the cochleae of early-postnatal *Eps8*^*−/−*^ pups (P1–P2) is able to restore the biophysical characteristics of the MET current in IHCs that have normal hair bundle morphology.

### IHCs from *Eps8*^*−/−*^ mice transduced with Anc80L65-Eps8 mature into normal sensory receptors

**Figure 6:**
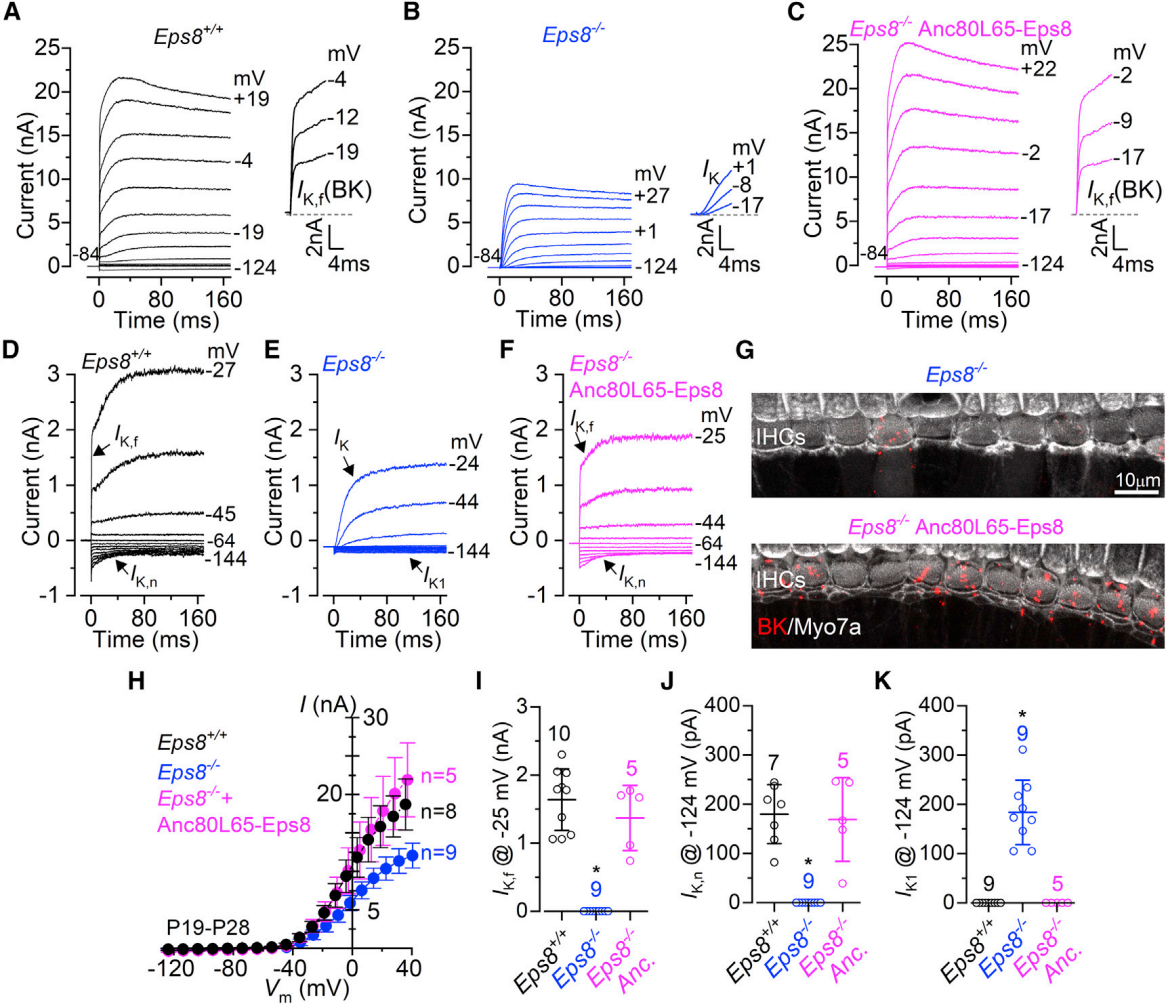
Rescue of basolateral membrane properties in adult IHCs from *Eps8*^*−/−*^ mice injected with Anc80L65-Eps8 (A–C) Current responses from IHCs of wild-type (*Eps8*^*+/+*^, P19: A), knockout (*Eps8*^*−/−*^, P28: B), and *Eps8*^*−/−*^ injected with Anc80L65-Eps8 (P22, C) mice. Current recordings were elicited by using depolarizing voltage steps (10-mV increments) from the holding potential of −84 mV to the various test potentials shown by some of the traces. The characteristic fast-activating BK current (*I*_K,f_) present in adult IHCs (A), which is better appreciated in the expanded timescale (see insets), was absent in *Eps8*^*−/−*^ mice (B) but rescued in cells transduced with Anc80L65-Eps8 (C). (D–F) Current responses from the same mouse models described in (A)–(C), but elicited using hyperpolarizing and depolarizing voltage steps (10-mV increments) from the holding potential of −64 mV to better highlight the inward currents. Adult IHCs from both *Eps8*^*+/+*^ (D) and *Eps8*^*−/−*^ mice injected with Anc80L65-Eps8 (F) express the characteristic KCNQ4-mediated delayed rectifier *I*_K,n_.^37^ IHCs from *Eps8*^*−/−*^ mice (E) retained the inward rectifier *I*_K1_, which is normally expressed only during pre-hearing stages.^36^ (G) Maximum intensity projections of confocal z stacks taken from the apical cochlear region of *Eps8*^*−/−*^ (top) and *Eps8*^*−/−*^ rescued (bottom) mice using antibodies against BK channels carrying *I*_K,f_ (red) and the hair-cell marker Myo7a (white). (H) Steady-state current-voltage curves obtained from IHCs of *Eps8*^*+/+*^ (P19–P28), *Eps8*^*−/−*^ mice (P20–P28) and *Eps8*^*−/−*^ injected (P22) mice. (I) Size of the fast-activating BK outward K^+^ current *I*_K,f_, which was measured at −25 mV and at 1 ms from the onset of the voltage step.^37^ (J) Size of the KCNQ4-mediated *I*_K,n_, which was measured as the difference between the peak and steady state of the deactivating inward current at −124 mV.^36^ (K) Size of the inward rectifier *I*_K1_ measured as the steady-state inward current at −124 mV.^36^ In (I)–(K), single cell value recordings (open symbols) are plotted behind the average data. Number of IHCs investigated is shown above the average data points. One-way ANOVA: p < 0.0001 (I–K) is indicated by the star symbol. For post test, see section “results”. Data are plotted as means ± SD.

The basolateral membrane properties of IHCs from *Eps8*^*−/−*^ mice have been shown to retain features of early stages of development, since they fail to express adult-like ion channels during maturation.^17^ We investigated, therefore, whether P19–P28 IHCs from *Eps8*^*−/−*^ mice transduced with the Anc80L65-Eps8 viral vector were able to mature into functional sensory receptors (Figures 6A–6K). One characteristic K^+^ current present in adult IHCs is *I*_K,f_, which is a rapid-activating, large-conductance Ca^2+^-activated K^+^ current carried by BK channels.^42^, ^43^, ^44^ Another mature-type K^+^ current in IHCs is that carried by KCNQ4 channels (*I*_K,n_).^37^^,^^45^ Both *I*_K,f_ and *I*_K,n_ were present in wild-type IHCs (Figures 6A and 6D), and both currents were absent in *Eps8*^*−/−*^ mice (Figures 6B, 6E, and 6G) as previously shown.^17^ IHCs from *Eps8*^*−/−*^ mice transduced with Anc80L65-Eps8, which showed normal-looking hair bundles, showed both currents (Figures 6C and 6F). BK channels were also normally localized near the neck region of the IHCs (Figure 6G) as previously shown.^46^ Over the entire voltage range investigated (−124 and +46 mV), the total K^+^ current was significantly smaller in non-transduced *Eps8*^*−/−*^ mice compared with both wild-type mice and *Eps8*^*−/−*^ mice transduced with the viral vector (p < 0.0001, two-way ANOVA; Figure 6H). When *I*_K,f_ and *I*_K,n_ were investigated in isolation, their size was not significantly different in IHCs from wild-type and *Eps8*^*−/−*^ mice transduced with Anc80L65-Eps8 (p = 0.3815 and p = 0.9357, respectively, two-way ANOVA, Tukey’s post test; Figures 6I and 6J). IHCs from *Eps8*^*−/−*^ mice not only lack *I*_K,f_ and *I*_K,n_ (Figures 6I and 6J) but also possess the inward rectifier *I*_K1_ (Figure 6K), a K^+^ current only present in immature hair cells.^36^ The immature current profile of IHCs from *Eps8*^*−/−*^ mice was also associated with the ability to elicit Ca^2+^-induced action potentials under current-clamp conditions (Figure S4), which is another biophysical characteristic of pre-hearing IHCs.^37^^,^^40^ By contrast, IHCs from wild-type and viral transduced *Eps8*^*−/−*^ mice elicit comparable graded-receptor potentials in response to depolarizing current injections, which is the normal behavior for adult cells.^42^ However, the resting membrane potentials were similar between the different mice investigated (p = 0.3487, one-way ANOVA; Figure S4), indicating that the contribution of the K^+^ currents active at rest (*I*_K,n_, wild-type and viral transduced *Eps8*^*−/−*^ mice; *I*_K1_, *Eps8*^*−/−*^ mice) was comparable.

**Figure 7:**
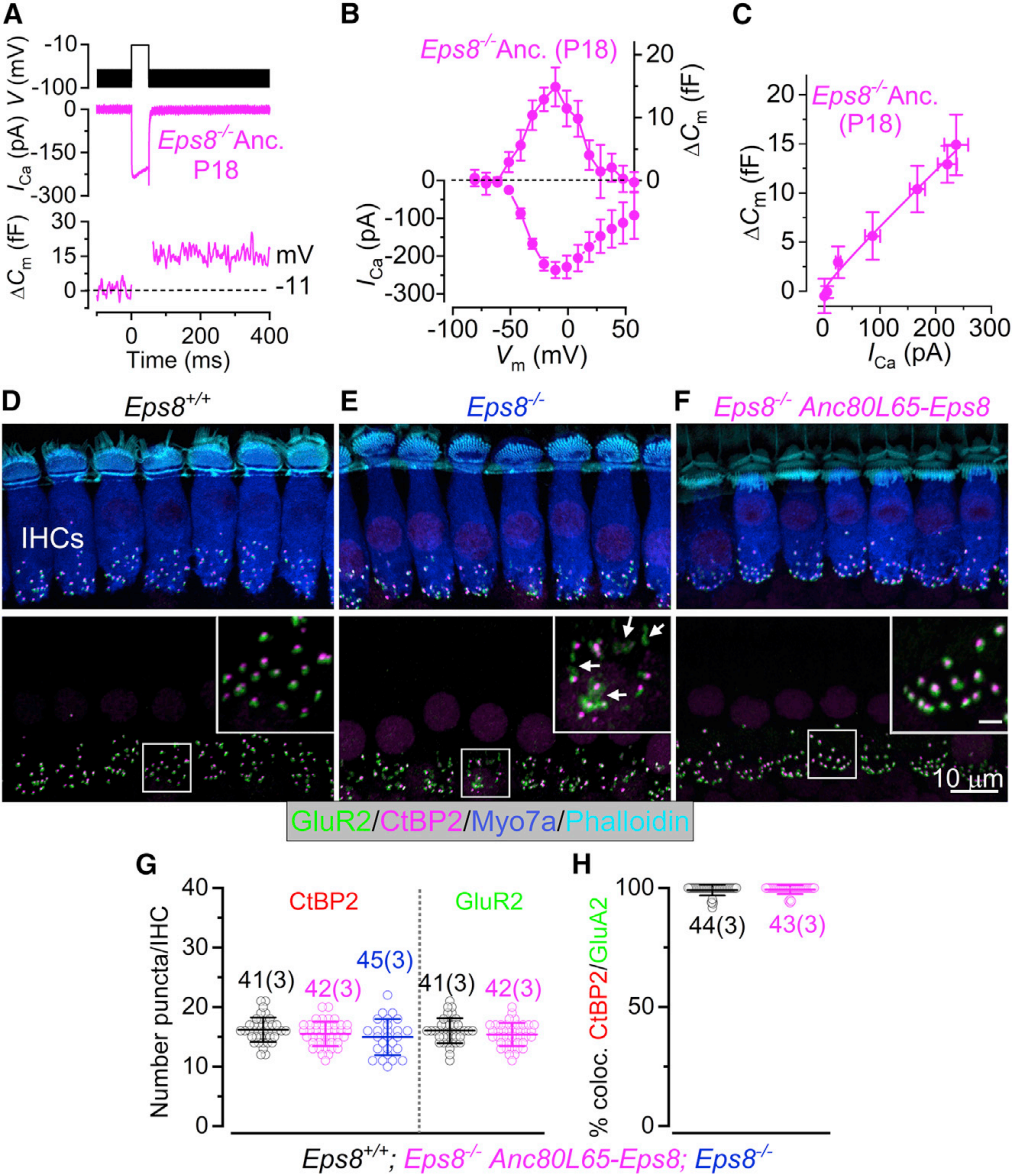
Exocytotic Ca^2+^ dependence and synaptic organization in *Eps8*^*−/−*^ mice transduced with Anc80L65-Eps8 (A) Calcium current (*I*_Ca_) and changes in membrane capacitance (Δ*C*_m_) recorded from a P18 IHC of an *Eps8*^*−/−*^ mouse transduced with Anc80L65-Eps8 at P1. Recordings were obtained in response to 50-ms voltage steps, in 10-mV increments, from the holding potential of −81 mV. For clarity, only maximal responses at −11 mV are shown. (B) Average *I*_Ca_ and ΔC_m_ curves over the entire voltage range tested from IHCs of *Eps8*^*−/−*^ mice transduced with Anc80L65-Eps8 at P1. (C) Synaptic transfer relations obtained by plotting ΔC_m_ against the corresponding *I*_Ca_ between −71 and −11 mV, showing that *Eps8*^*−/−*^ mice transduced with Anc80L65-Eps8 show a normal linear Ca^2+^ dependence of exocytosis. Data were approximated using a power function $\Delta C_{\text{m}}=c{I_{\text{Ca}}}^{n}$, where *c* is a scaling coefficient and the exponent *n* is the power. (D–F) Maximum intensity projections of confocal z stacks of apical-coil IHCs from >P20 *Eps8*^*+/+*^ (D), *Eps8*^*−/−*^ (E), and *Eps8*^*−/−*^ transduced with Anc80L65-Eps8 at P1 (F) mice. IHCs were labeled with antibodies against CtBP2 (ribbon synaptic marker: red) and GluR2 (post-synaptic marker: green). Myosin 7a (Myo7a) was used as the IHC marker (blue) and phalloidin as the stereociliary marker (Cyan). Insets show a magnified view of the co-localization between the CtBP2 and GluR2. Note that, in the *Eps8*^*−/−*^ IHCs (E), but not control (D) and transduced (F) cells, the post-synaptic marker GluR2 shows a diffuse distribution (see arrows in the magnified view of E). Scale bars in the insets represent 2 μm. (G) Number of CtBP2 and GluA2 puncta in IHCs from *Eps8*^*+/+*^ (black) and *Eps8*^*−/−*^ (blue) transduced with Anc80L65-Eps8 (magenta) mice. Please note that, for *Eps8*^*−/−*^ mice, we could not quantify the GluR2 labeling because it shows a very diffused profile. (H) Number of co-localized CtBP2 and GluR2 puncta in *Eps8*^*+/+*^ (black) and *Eps8*^*−/−*^ transduced with Anc80L65-Eps8 (magenta) mice. Data in (G) and (H) are plotted as mean values and individual counts are shown as open symbols. Numbers above or below the data represent the IHCs (and mice) used for each genotype. Average values are mean ± SD.

We then investigated exocytosis from the ribbon synapses of IHCs transduced with Anc80L65-Eps8 (Figures 7A–7C). Exocytosis was estimated by measuring increases in cell membrane capacitance (*ΔC*_m_) following depolarizing voltage steps that activate the Ca^2+^ current (see section “materials and methods”). A previous study has shown that, in *Eps8*^*−/−*^ mice, IHCs retain a high-order Ca^2+^ dependence of glutamate-containing vesicle release,^17^ which is normally only present during pre-hearing stages of development in apical-coil mouse IHCs.^47^^,^^48^ We found that IHCs from *Eps8*^*−/−*^ mice transduced with Anc80L65-Eps8 at P1 develop a normal linear Ca^2+^-dependent exocytosis, as indicated by the nearly unitary exponent resulting from the fit with the power function (0.94 ± 0.23, n = 5; Figure 7C). This power value was not significantly different to that previously measured in *Eps8*^*+/+*^ mice (1.17 ± 0.26, n = 5, p = 0.8872), but significantly different from *Eps8*^*−/−*^ mice (3.35 ± 1.31, n = 5, p = 0.0010, Tukey’s post test, one-way ANOVA).^17^

To investigate the synapses between the IHCs and the spiral ganglion afferent terminals, which are key to proper sound encoding, we used antibodies to label the presynaptic ribbon protein RIBEYE (CtBP2) and the post-synaptic AMPA-type glutamate receptor GluR2.^49^^,^^50^ Compared with wild-type *Eps8*^*+/+*^ hair cells, we found that both CtBP2 and GluR2 puncta were present at the pre- and post-synaptic sites, respectively, in IHCs from both the *Eps8*^*+/+*^ mice and the *Eps8*^*−/−*^ mice transduced with Anc80L65-Eps8 (Figures 7D–7F). The number of CtBP2 puncta in IHCs was not significantly different between *Eps8*^*+/+*^, *Eps8*^*−/−*^, and *Eps8*^*−/−*^ mice transduced with Anc80L65-Eps8 (p = 0.3839, one-way ANOVA; Figure 7G). Post-synaptic GluR2 labeling was organized in its characteristic puncta-like distribution in *Eps8*^*+/+*^ mice (Figure 7D), but we found that *Eps8*^*−/−*^ mice showed a more diffuse organization of GluR2, which prevented the reliable quantification of discrete puncta (Figure 7E, arrows). Anc80L65-Eps8 restored the normal puncta-like organization of GluR2 in the *Eps8*^*−/−*^ mice (Figure 7F). A similar diffuse distribution of GluR2 was recently described in *Tmc1;Tmc2* double-knockout mice, in which the MET current is absent,^51^ similar to the *Eps8*^*−/−*^ mice (Figure 7E; see also Zampini et al.^17^). The number of GluR2 puncta was not significantly different between the non-transduced *Eps8*^*+/+*^ mice and the *Eps8*^*−/−*^ mice transduced with Anc80L65-Eps8 (p = 0.1367, t test; Figure 7G) and showed statistically similar co-localization with CtBP2 puncta-like organization (p = 0.5877, t test; Figure 7H).

Together, these findings show that virally transduced *Eps8*^*−/−*^ IHCs can produce normal-looking hair bundles and can fully mature into functional sensory receptors.

### OHC stereocilia from *Eps8*^*−/−*^ mice transduced with AAV do not attach to the tectorial membrane

OHC stereocilia are attached to the tectorial membrane and react against this structure in response to sound stimuli. This connection can be confirmed by the presence of imprints of the tallest stereocilia in the tectorial membrane or a protein that connects the stereocilia to the tectorial membrane, such as stereocilin.^52^ Labeling of whole-mount cochlear preparations from P22 wild-type (C57BL/6N) mice with antibodies to stereocilin revealed puncta primarily localized at the tips of the tallest row of stereocilia, with some staining also associated with the stereocilia of the shorter rows (Figure S5A). In contrast, in *Eps8*^*−/−*^ mice, in which the stereocilia are shorter, stereocilin is no longer present to the tips of the taller stereocilia (Figure S5B). In *Eps8*^*−/−*^ mice in which the cochleae were injected with Anc80L65-Eps8, and OHC stereocilia showed near-normal growth, stereocilin nevertheless failed to localize to the tips of the tallest row of stereocilia (Figure S5C). Consistent with these observations, examination of the underside of the tectorial membrane (TM) in preparations stained with wheat-germ agglutinin revealed that imprints were only present in TMs derived from wild-type cochleae, and not from those of *Eps8*^*−/−*^ mice or Anc80L65-Eps8 transduced *Eps8*^*−/−*^ mice (Figures S5D–S5F). These findings show that the even the few OHCs that were showing normal-looking hair bundles were unable to interact with the TM, thus explaining the lack of DPOAEs in these mice (Figure S3).

### Injection of Anc80L65-Eps8 into the cochleae of adult mice fails to rescue Eps8 localization at the tip of stereocilia

**Figure 8:**
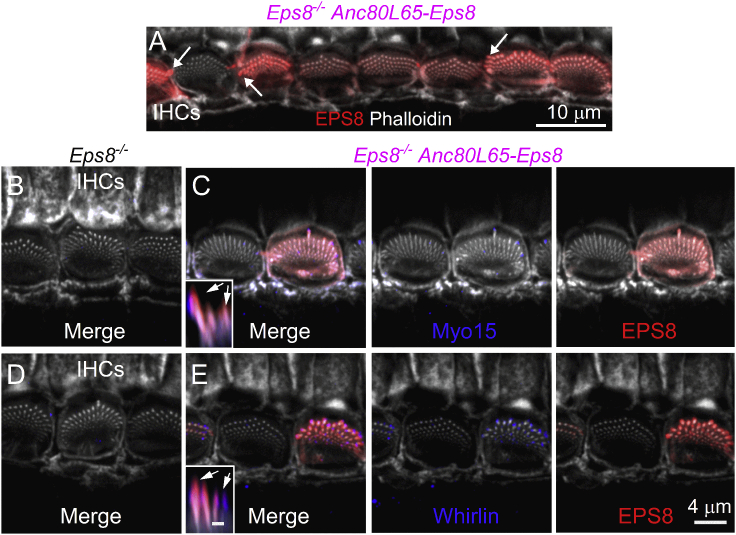
Hair bundles in IHCs transduced with Anc80L65-Eps8 in the adult cochlea (A) Confocal images of the hair bundles from IHCs of 3-month-old *Eps8*^*−/−*^ mice transduced with Anc80L65-EPS8 at P20. Adult cochleae were immunostained for EPS8 (red) and stereocilia are labeled with phalloidin (green). Note that transduced IHCs still show several rows of stereocilia, most of which remained short. Moreover, EPS8 is no longer localized at the tip of the taller stereocilia. (B–E) Confocal images using the same experimental conditions described in (A), but using antibodies against MYO15 (B and C) and WHIRLIN (D and E), in addition to EPS8. Note that the diffuse staining of all the proteins, similar to the labeling seen in basal-coil IHCs from *Eps8*^*−/−*^ mice transduced with Anc80L65-EPS8 at P1–P2. Scale bar in the inset of (E), which also applies for (C), represents 1 μm.

We tested the ability of the AAVs to transduce cochlear hair cells of older mice. Despite the successful transduction of Acn80L65-Eps8 in adult *Eps8*^*−/−*^ mice (injected at P19–P20), transduced IHCs failed to localize EPS8 to the tips of their taller stereocilia (Figure 8A). Instead, EPS8 appears to be present along the entire length of these stereocilia, which predominantly remained short. A few hair cells still possessed extra rows of short stereocilia normally associated with immature hair bundles. A few stereocilia within bundles of transduced hair cells did elongate; however, these still showed a diffuse labeling of EPS8 (Figure 8A, arrows). Even though the bundle structure of the IHCs from *Eps8*^*−/−*^ mice transduced with Acn80L65-Eps8 was similar to that of IHCs from *Eps8*^*−/−*^ mice, they were able to localize MYO15 and WHIRLIN to their stereocilia (Figures 8C–8E). However, similar to the distribution of EPS8 (Figure 8A), or that of WHIRLIN in basal-coil IHCs (Figures 4J and 4K), these proteins showed a more diffuse distribution along the length of several rows of stereocilia (Figures 8C–8E, arrows).

## Discussion

The correct development of cochlear hair cells into fully mature sensory receptors is a tightly regulated process requiring the interaction of several proteins, including the actin-regulatory protein EPS8.^16^^,^^17^ The absence of EPS8 has been shown to halt the normal development of hair cells, especially that of the IHCs, holding them at pre-hearing immature stages, preventing them from processing acoustic stimuli.^17^ We demonstrated that exogenous delivery of EPS8 using the AAV vector Anc80L65 in *Eps8*^*−/−*^ in P1–P2 pups *in vivo* can restore the functional characteristics of transduced apical-coil hair cells in adult mice. Exogenous EPS8 was able to rescue not only the normal staircase structure of the stereociliary bundle and have normal mechanoelectrical transduction but also the expression of adult-like basolateral ion channels and the distribution of post-synaptic glutamate receptors. Despite the full recovery of some hair cells, we observed very little to no recovery of hearing function. This may be due to the failure of transduced OHC bundles to attach to the TM. We also found that transduced hair cells from P3 apical coil or P1–P2 basal coil of the cochlea maintained the immature hair bundle structure characteristic of *Eps8*^*−/−*^ mice, and did not localize EPS8 to stereocilia tips, similar to when exogenous delivery of Eps8 was performed in P19–P20 *Eps8*^*−/−*^ mice with functionally mature IHCs. We propose that exogenous gene augmentation using AAV vectors *in vivo* is a suitable strategy to rescue complex morphological and functional defects present in hair cells of a mouse model of recessive deafness. However, by postnatal ages, most of the hair cells of *Eps8*^*−/−*^ mice may have matured or accumulated damage beyond the point for certain morphological or physiological features to be repaired. Therefore, we propose that, for genes that regulate early hair-cell development (i.e., embryonic onset of function), gene-based therapeutic approaches may need to be performed *in utero* in order to recover hearing function.

### *In vivo* delivery of AAV-Eps8 in early-postnatal *Eps8*-deficient mice restores the function of hair cells

The precise staircase structure of the stereociliary bundles of cochlear hair cells develops primarily during pre-hearing stages (prior to ∼ P12 in mice) through a tightly controlled process of elongation and thickening, as well as an elimination of redundant stereocilia.^5^^,^^53^ This precise regulation of the stereociliary actin cytoskeleton is defined by several proteins, most of which are already expressed in the emerging stereocilia during embryonic stages of development,^23^^,^^33^^,^^34^ and their absence leads to largely undeveloped hair bundles in both IHCs and OHCs, and consequent hearing loss. EPS8 is one of these key proteins since it is expressed from late embryonic stages of development in mice and it is required for the initial elongation of the immature stereociliary bundles of hair cells, as well as the reabsorption of the supernumerary stereocilia.^16^^,^^17^ Interestingly, MYO15, WHIRLIN, and BAIAP2L2 did not localize to stereocilia tips in *Eps8*^*−/−*^ mice, suggesting EPS8 is critical for the proper transport and/or retention of these proteins at stereocilia tips. In *Eps8*^*−/−*^ mice, the immature hair bundle structure causes a wide range of cellular dysfunctions, including a very small or absent resting MET current (Figure 5), possibly due to a reduced tension at the tip links connecting adjacent stereocilia that normally keep the MET channel partially open at rest.^7^ This resting depolarizing MET current normally drives the resting firing activity in the auditory fibers of the adult cochlea. However, the MET apparatus is fully functional before hearing onset in mice,^38^^,^^54^^,^^55^ and the resting MET current has been shown to be required for the normal maturation of the IHC biophysical properties^40^ and their afferent synapses.^51^ We also observed this in *Eps8*^*−/−*^ mice (Figures 6 and 7). As such, EPS8 regulates the physiological and morphological maturation of IHCs and in its absence IHCs remain arrested at very early stages of development. All these morphological and physiological defects lead to profound deafness in *Eps8*^*−/−*^ mice.

When exogenous EPS8 was delivered to *Eps8*^*−/−*^ pups (P1–P2) via Anc80L65 *in vivo*, we found that some transduced apical-coil hair cells were able to develop normal bundles, including the reabsorption of the extra numeral stereociliary rows (Figure 2). These normal-looking hair bundles generated an MET current in pre-hearing hair cells that was indistinguishable from that of wild-type mice, and showed a normal distribution pattern of EPS8, MYO15, and WHIRLIN, including the presence of BAIAP2L2 at the shorter rows (Figures 3 and 4). The recovery of the MET current meant that some transduced IHCs from *Eps8*^*−/−*^ mice were able to progress with their normal development and acquire the biophysical basolateral membrane characteristics and morphological features of fully mature sensory receptors (Figures 6 and 7).

We found, however, that only some apical-coil hair cells from Anc80L65-Eps8 transduced *Eps8*^*−/−*^ mice were able to fully recover (about 20%), with several only showing an intermediate phenotype exhibiting hair bundles containing several rows of both short and elongated stereocilia (Figure 2). Interestingly, transduced apical-coil hair cells from *Eps8*^*−/−*^ mice injected at P3 and basal-coil hair cells from *Eps8*^*−/−*^ mice transduced at P1–P2 failed to recover any normal hair bundle structure, or to localize EPS8 and WHIRLIN to the tips of the taller stereocilia (Figure 4). A similar profile was also identified when EPS8 augmentation with AAV was performed in P19–P20 mice, with EPS8, MYO15, and WHIRLIN labeling the entire length of the stereocilia (Figure 8). Together, these results indicate a role for EPS8 in the proper targeting of these tip-complex proteins to stereocilia tips, which is perhaps counterintuitive given MYO15 is the motor protein responsible for transporting these proteins to stereocilia tips.

### AAV gene delivery as a therapy to restore the function of early-onset genes in the mouse cochlea

More than 100 genes have now been linked to hereditary hearing impairment (https://hereditaryhearingloss.org/), most of which are expressed in the sensory hair cells of the mammalian cochlea.^2^ During the last decade or so, several AAV vectors have been used to transduce exogenous genes *in vivo* into dysfunctional hair cells of different mouse models of hereditary HL.^2^^,^^56^^,^^57^ Although the success of this approach was limited, at least initially, recent development in the viral capsids used for gene augmentation *in vivo* (Anc80L65,^28^ AAV-S,^58^ AAV9-PHP.B^59^) has resulted in more efficient hair-cell transduction and functional recovery. In agreement with these previous reports, we found that Anc80L65 leads to a high transduction rate, at least in the apical coil of the cochlea, which was the region used for the *ex vivo* functional recovery experiments. It is therefore unlikely that a low infection efficiency of Anc80L65 is responsible for the very little or no functional recovery of low-frequency hearing in *Eps8*^*−/−*^ mice injected with the exogenous EPS8. It is also worth noting that our surgery procedure did not cause any damage to the cochlea since wild-type mice subjected to the same procedure as *Eps8*^*−/−*^ mice showed normal auditory functions at adult ages.

One possible explanation for the absence of hearing recovery is that, at the time we performed the AAV gene delivery (primarily P1), most of the hair cells are at a point in development such that they have either matured or accumulated damage beyond the point of repair using therapeutic gene delivery. This is because EPS8 exerts a key role in bundle growth from embryonic stages of development. In fact, the transduction of Anc80L65-Eps8 in hair cells from the basal-coil region of the cochlea of *Eps8*^*−/−*^ mice, which are known to be approximately 2–3 days ahead in terms of development compared with apical cells,^37^^,^^38^ failed to rescue their immature-like hair bundles. Similar findings were also seen when attempting to correct mature hair cells from P19–P20 *Eps8*^*−/−*^ mice. Evidence of a critical period for therapeutic gene delivery in the cochlea seems to be consistent with the published literature. Most of the successful functional recovery of mouse models for hereditary monogenic hearing impairment has involved genes that primarily contribute to the development and/or function of the postnatal mouse cochlea (e.g., *Tmc1*,^60^^,^^61^ *Vglut3*,^62^ *Otof*^63^^,^^64^). Gene-based therapy targeting proteins that are directly involved in the initial growth of the stereociliary bundles of cochlear hair cells has mainly focused on harmonin, whirlin, and clarin-1, which lead to different forms of Usher syndromes when mutated (Usher 1C, Usher 2, and Usher 3A, respectively).^65^^,^^66^ Harmonin is present at the tip of the stereociliary bundle from about P0 onward,^67^ and AAV gene augmentation at P1 with Anc80L65-harmonin injected in the cochlea of a mouse model for Usher 1C led to a reasonable hearing rescue in the low-frequency (<16 Hz) but not high-frequency regions.^68^ Clarin-1 and whirlin are expressed in hair bundles just before birth,^32^^,^^69^ and AAV gene delivery leads to a very limited or no recovery of hearing in corresponding mouse models for Usher 3A (clarin-1, ABR threshold ∼90 dB only at about 10 kHz)^70^ and Usher 2 (whirlin: ABR threshold >80 dB only at 8 kHz^71^ or deaf^72^). We speculate that, for genes involved in the early stages of morphological and functional development of cochlear hair cells, there is a critical window for successful therapeutic gene delivery for treating hearing loss, and, in some cases (e.g., EPS8 and some Usher proteins), *in utero* delivery of genes via AAVs may be required as previously performed for other genes.^73^^,^^74^ Alternatively, it is possible that co-expression of other proteins or co-administration of small molecules that can expand this critical window will increase success in restoring hearing function.

## Materials and methods

### Ethics statement

The animal work was licensed by the UK Home Office under the Animals (Scientific Procedures) Act 1986 (PPL_PCC8E5E93) and was approved by the University of Sheffield Ethical Review Committee (180626_Mar). *Eps8* knockout mice (*Eps8*^*−/−*^) on a C57BL/6N background were breed as either *Eps8*^*+/−*^ × *Eps8*^*−/−*^ or *Eps8*^*−/−*^ × *Eps8*^*−/−*^. Both males and females where used for this study. For *ex vivo* experiments, mice were killed by cervical dislocation followed by decapitation. For *in vivo* ABRs and DPOAEs, mice were anesthetized using intraperitoneal injection of ketamine (100 mg/kg body weight, Fort Dodge Animal Health, Fort Dodge, USA) and xylazine (10 mg/kg, Rompun 2%, Bayer HealthCare, NY, USA). At the end of the *in vivo* recordings, mice were either culled by cervical dislocation or recovered from anesthesia with intraperitoneal injection of atipamezole (1 mg/kg). For *in vivo* gene delivery, mice were anesthetized with isoflurane (2.5%) under oxygenation (0.8%). Mice under recovery from anesthesia were returned to their cage, placed on a thermal mat, and monitored over the following 2–5 h.

### AAV production

The original Eps8 cDNA was a gift from Giorgio Scita and Andrea Disanza. The EGFP-mouse Eps8-beta-globin-polyA coding cDNA was synthesized using PCR and was cloned into the pAAV vector (with cytomegalovirus [CMV] promoter) using the SacI and XbaI restriction enzymes sites. The FLAG-tag-Eps8 coding cDNA was synthesized using PCR and was cloned into the pAAV vector (with CMV promoter) using the EcoRI and HindIII restriction enzymes sites. All constructs have been verified by multiple restriction enzyme digestions and sequencing. AAV-Anc80L65.CMV.FLAG-mEps8.bGH (Anc80L65-Eps8) and AAV-Anc80L65.CMV.PI.eGFP.WPRE.bGH (Anc80L65-GFP) were produced by the Penn Vector Core at the University of Pennsylvania (USA). The pAAV-Anc80L65 plasmid was purchased from Addgene (92307).

### AAV gene delivery *in vivo* for both pups and adult mice

The surgical protocol used for AAV injection into the cochlea of P1–P3 and adult mice was performed under anesthesia (see section “ethics statement”) and body temperature was maintained using a heating mat. The right ear was accessed via an incision just below the pinna as previously described.^62^^,^^75^ When the RWM was identified, it was gently punctured with a borosilicate pipette. This was followed by the injection of the AAV into the cochlea (pressure controlled by mouth) of 1 μL of Anc80L65-GFP (2.58 × 10^13^ vg/mL) or Anc80L65-Eps8 (8.90 × 10^12^ vg/mL), which was the maximal titer achievable from the supplied AAV. Following the injection, the pipette was retracted from the RWM and the wound was closed with veterinarian glue.

### Tissue preparation for *ex vivo* recordings

Patch clamp recordings were performed from hair cells within the 9–12 kHz region of the cochlear apical coil.^76^ Cochleae were dissected out in extracellular solution composed of (in mM) 135 NaCl, 5.8 KCl, 1.3 CaCl_2_, 0.9 MgCl_2_, 0.7 NaH_2_PO_4_, 5.6 D-glucose, and 10 HEPES-NaOH. Sodium pyruvate (2 mM), amino acids and vitamins were added from concentrates (Thermo Fisher Scientific, UK). The pH was adjusted to 7.48 with 1 M NaOH, and the final osmolality of the solution was measured (∼308 mmol kg^−1^). The dissected apical coil was then transferred to a microscope chamber and immobilized via a nylon mesh attached to a stainless-steel ring. The chamber (volume 2 mL) was perfused from a peristaltic pump and mounted on the stage of an upright microscope (Olympus BX51, Japan) with Nomarski DIC optics. For electrophysiology, the hair bundle structure of the hair cells was assessed using a 60× water immersion objective, and an additional 2× magnification prior the eyepiece (15×). The microscope chamber was continuously perfused with extracellular solution by a peristaltic pump (Cole-Palmer, UK).

### Whole-cell electrophysiology

Patch-clamp recordings were performed at room temperature (20°C –24°C) using an Optopatch amplifier (Cairn Research, UK) as previously described.^25^^,^^77^^,^^78^ Patch pipettes were pulled from soda glass capillaries and had a typical resistance in extracellular solution of 2–3 MΩ. The patch pipette intracellular solution contained (in mM) 131 KCl, 3 MgCl_2_, 1 EGTA-KOH, 5 Na_2_ATP, 5 HEPES-KOH, and 10 Na-phosphocreatine (pH was adjusted with 1 M KOH to 7.28; 294 mmol kg^−1^). Data acquisition was controlled by pClamp software using Digidata 1440A (Molecular Devices, USA). To reduce the electrode capacitance, patch electrodes were coated with surf wax (Mr. Zog’s SexWax, USA). Recordings were low-pass filtered at 2.5 kHz (eight-pole Bessel), sampled at 5 kHz, and stored on a computer for offline analysis (Clampfit, Molecular Devices; Origin 2020; OriginLab, USA). For voltage-clamp experiments, membrane potentials were corrected offline for the residual series resistance *R*_s_ after compensation (usually 80%) and the liquid junction potential (LJP) of −4 mV, which was measured between electrode and bath solutions. Holding currents were plotted as zero current to allow a better comparison between recordings. Voltage-clamp protocols are referred to a holding potential of −84 mV or −64 mV depending on the protocol used.

For MET current recordings, the bundles of hair cells were displaced using a fluid jet from a pipette driven by a 25-mm diameter piezoelectric disc.^39^^,^^40^ The pipette was pulled from borosilicate glass to a final overall length of 5.3–5.5 cm. The fluid jet pipette tip had a diameter of 8–10 μm and was positioned near the hair bundles to elicit a maximal MET current. Mechanical stimuli were applied as 50-Hz sinusoids (filtered at 1 kHz, eight-pole Bessel). Prior to the positioning of the fluid jet by the hair bundles, any steady-state pressure was removed by monitoring the movement of debris in front of the pipette. The use of the fluid jet allows for the efficient displacement of the hair bundles in both the excitatory and inhibitory directions, which is essential to perform reliable measurements of the resting open probability of the MET channels.

Real-time changes in membrane capacitance (Δ*C*_m_) were measured as previously described.^77^^,^^79^ Briefly, Δ*C*_m_ was measured using a 4-kHz sine wave (13 mV RMS) applied to hair cells from a holding potential of −81 mV and was interrupted for the duration of the voltage step. The capacitance signal from the Optopatch was amplified (×50), filtered at 250 Hz, sampled at 5 kHz, and measured by averaging the *C*_m_ traces after the voltage step (around 200 ms) and subtracting from pre-pulse baseline. Δ*C*_m_ was recorded in the presence of K^+^ channel blockers TEA (30 mM), 4-AP (15 mM), and linopirdine (80 μM) in the extracellular solution.

### ABRs

Following the onset of anesthesia (see section “ethics statement”) and the loss of the retraction reflex with a toe pinch, mice were placed in a soundproof chamber (MAC-3 acoustic chamber, IAC Acoustic, UK). Male and female mice were placed on a heated mat (37°C) with the animal’s pinna being positioned at a distance of 10 cm from the loudspeaker. Two subdermal electrodes were placed under the skin behind the pinna of each ear (reference and ground electrode), and one electrode half-way between the two pinna on the vertex of the cranium (active electrode) as previously described.^80^ Sound stimuli were delivered to the mouse ear by a loudspeaker (MF1-S, Multi Field Speaker, Tucker-Davis Technologies, USA), which was calibrated with a low-noise microphone probe system (ER10B+, Etymotic, USA). Experiments were performed using customized software^80^ driving an RZ6 auditory processor (Tucker-Davis Technologies). ABR thresholds, which were delivered as white-noise clicks and pure-tone stimuli of frequencies at 6, 12, 18, 24, 30, 36, and 42 kHz, were defined as the lowest sound level where any recognizable feature of the waveform was visible. Stimulus sound pressure levels were up to 95 dB SPL, were presented in steps of 5-dB SPL (averaged over 256 repetitions). Tone bursts were 5 ms in duration with a 1-ms on/off ramp time presented at a rate of 42.6/s.

### DPOAEs

DPOAEs were used to assess OHC function *in vivo* by the synchronous presentation of two stimulus tones (primaries f1 and f2). DPOAEs were recorded at 2f1-f2 in response to primary tones f1 and f2, where f2/f1 = 1.2. The f2 level (L2) was set from 20 to 80 dB (maximum level set for our system) in 10-dB increments, and the f1 level (L1) was set equal to L2. Frequency pairs of tones between f2 = 6.5 kHz and f2 = 26.3 kHz were presented directly into the left ear canal of mice by means of a coupler, which was connected to two calibrated loudspeakers using 3-cm plastic tubes (MF1-S, Multi Field Speaker, Tucker-Davis Technologies, USA).

Recordings were performed in a soundproof chamber (MAC-3 Acoustic Chamber, IAC Acoustic, UK) and the emission signals were recorded by a low-noise microphone (ER10B+, Etymotic Research, USA) connected to the coupler mentioned above. Experiments were performed using BioSigRZ software driving an RZ6 auditory processor (Tucker-Davis Technologies). The DPOAE thresholds were defined by the minimal sound level where the DPOAEs were above the standard deviation (SD) of the noise. The determined DPOAE thresholds were plotted against the geometric mean frequency of f1 and f2. Stimulus sound pressure levels were up to 80 dB SPL, presented in steps of 10 dB. The response signal was averaged over 500 repetitions.

### SEM

For SEM, the dissected mouse cochleae were initially fixed by a very gentle intralabyrinthine perfusion using a 100-μL pipette tip through the round window. The fixative contained 2.5% glutaraldehyde in 0.1 M sodium cacodylate buffer plus 2 mM CaCl_2_ (pH 7.4). Following perfusion, the cochleae were immersed in the above fixative for 2 h at room temperature. After the fixation, the organ of Corti was exposed by removing the bone from the apical coil, and the cochleae, then incubated in solutions of saturated aqueous thiocarbohydrazide (20 min) alternating with 1% osmium tetroxide in cacodylate buffer (2 h) twice (the OTOTO technique).^81^ The cochleae were then dehydrated through an ethanol series and critical point dried using CO_2_ as the transitional fluid (Leica EM CPD300) and mounted on specimen stubs using conductive silver paint (Agar Scientific, Stansted, UK). The apical coil of the organ of Corti was examined at 10 kV using a Tescan Vega3 LMU scanning electron microscope.

### Immunofluorescence microscopy

The majority of this work was performed by fixing the inner ear with 4% paraformaldehyde in phosphate-buffered saline (PBS, pH 7.4) for 20 min at room temperature. Cochleae were washed three times in PBS for 10 min and fine dissected. Samples were incubated in PBS supplemented with 5% normal goat or horse serum and 0.5% Triton X-100 for 1 h at room temperature. The samples were immunolabelled with primary antibodies overnight at 37°C, washed three times with PBS, and incubated with the secondary antibodies for 1 h at 37°C. Antibodies were prepared in 1% serum and 0.5% Triton X-100 in PBS. F-actin was stained with Texas Red-X phalloidin (1:400, Thermo Fisher, T7471) or Alexa Fluor 488 phalloidin (1:400, Thermo Fisher, A12379) in the secondary antibody solution. Primary antibodies were mouse-IgG1 anti-Eps8 (1:1,000, BD Biosciences, 610143), rabbit-IgG anti-BAIAP2L2 (1:500, Atlas Antibodies, HPA003043), mouse-IgG1 anti-BK channel (1:200, NeuroMab, 75–408), rabbit-IgG anti-MYO7a (1:500, Proteus Biosciences, 25–6790), anti-MYO15 and anti-WHIRLIN (1:400 and 1:200, respectively, gift from Dr. Thomas Friedman, NIH, USA), mouse anti-CtBP2 (1:200, Biosciences, #612044), mouse anti-GluR2 (1:200, Millipore, MAB397), and rabbit-anti-FLAG (1:200, Cell Signaling, #14793). Secondary antibodies were species appropriate Alexa Fluor secondary antibodies. Samples were mounted in VECTASHIELD (H-1000). The images from the apical cochlear region (8–12 kHz) were captured with Nikon A1 confocal microscope equipped with Nikon CFI Plan Apo 60× oil objective or a Zeiss LSM 880 Airyscan equipped with Plan-Apochromat 63× Oil DIC M27 objective for super-resolution images of hair bundles. Both microscopes are part of the Wolfson Light Microscope Facility at the University of Sheffield. Image stacks were processed with Fiji ImageJ software (https://imagej.net/Fiji).^82^

For the immunostaining to investigate OHC insertion into the TM, the inner ear was fixed with 4% paraformaldehyde in PBS (pH 7.4) for 1 h at room temperature. Cochleae were then decalcified in 0.5 M EDTA for 3 days at 4°C, washed in PBS, and dissected as previously described.^83^ These were sub-dissected by iterative trimming with a razor blade, and the tectorial membrane (TM) segments carefully peeled off from the tissue pieces with a pair of fine forceps. These TM pieces were transferred into dishes containing PBS with 10% horse serum and 0.1% Triton X-100 (PBS/HS/TX) to which FITC-conjugated wheat-germ agglutinin (FL-1021, Vector Laboratories) was added at a dilution of 1:1,000. These were incubated overnight at 4°C with gentle agitation, washed in PBS containing 0.1% TX (PBS/TX), and mounted on glass slides in Vectashield with the underside of the TM facing upward. Pieces of organ of Corti with the TMs removed were transferred to PBS/HS/TX for 1 h, followed by PBS/HS/TX containing rabbit anti-stereocilin (1:150, gift from Dr. Saaid Saffieddine, Institut Pasteur, France) and 1:1,000 Phalloidin-Atto 488 (49049, Sigma-Aldrich). The tissue was then incubated overnight at 4°C with gentle agitation, washed three times in PBS/TX and stained for 3 h in Alexa 555 donkey anti-rabbit (A31572, Invitrogen), washed three times in PBS/TX, and mounted in Vectashield (Vector Laboratories). Super-resolution scans of these preparations were obtained on a Zeiss LSM 880 with an Airyscan detector, using a 63×1.4 NA oil-immersion objective. Z-projections were created using Imaris Viewer 9.8.0.

### Measurements of stereocilia dimensions

Image analysis and any further processing for visualization was performed using Fiji. Airyscan processed z stacks were resliced along the y or x axis using the Reslice feature. The tallest stereocilia of each bundle was identified and measured in the phalloidin channel using the Segmented Line tool of ImageJ. Bundles were considered EPS8 positive if EPS8 puncta were visible at the tips of stereocilia within the bundle. Non-transduced hair cells within the same field were used to measure EPS8-negative stereocilia height. For these measurements, IHCs were selected based on the presence of a tip-localized EPS8 signal, regardless of the amount of improved bundle height apparent prior to quantification. Only the tallest stereocilia of each IHC was measured.

### Statistical analysis

Statistical comparisons of means were made by Student’s two-tailed t test or, for multiple comparisons, analysis of variance (one-way or two-way ANOVA followed by a suitable post test); p < 0.05 was selected as the criterion for statistical significance. Only mean values with a similar variance between groups were compared. Average values are quoted in text and figures as mean ± SD.

### Data availability statement

The data that support the findings of this study are available from the corresponding authors upon reasonable request.

## Supplemental information

Document S1. Figures S1–S6

Document S2. Article plus supplemental information
