# Supplementary material for: AAV-mediated rescue of Eps8 expression in vivo restores hair-cell function in a mouse model of recessive deafness
Source: Mol Ther Methods Clin Dev. 2022 Jul 31;26:355–70. doi: 10.1016/j.omtm.2022.07.012 (PMC9382420; doi:10.1016/j.omtm.2022.07.012)
Supplement: Document S1. Figures S1–S6 [file mmc1.pdf]

OMTM, Volume *26*

Supplemental information

AAV-mediated rescue of *Eps8* expression *in vivo* restores hair-cell function in a mouse model of recessive deafness

Jing-Yi Jeng, Adam J. Carlton, Richard J. Goodyear, Colbie Chinowsky, Federico Ceriani, Stuart L. Johnson, Tsung-Chang Sung, Yelena Dayn, Guy P. Richardson, Michael R. Bowl, Steve D.M. Brown, Uri Manor, and Walter Marcotti

Figure S1

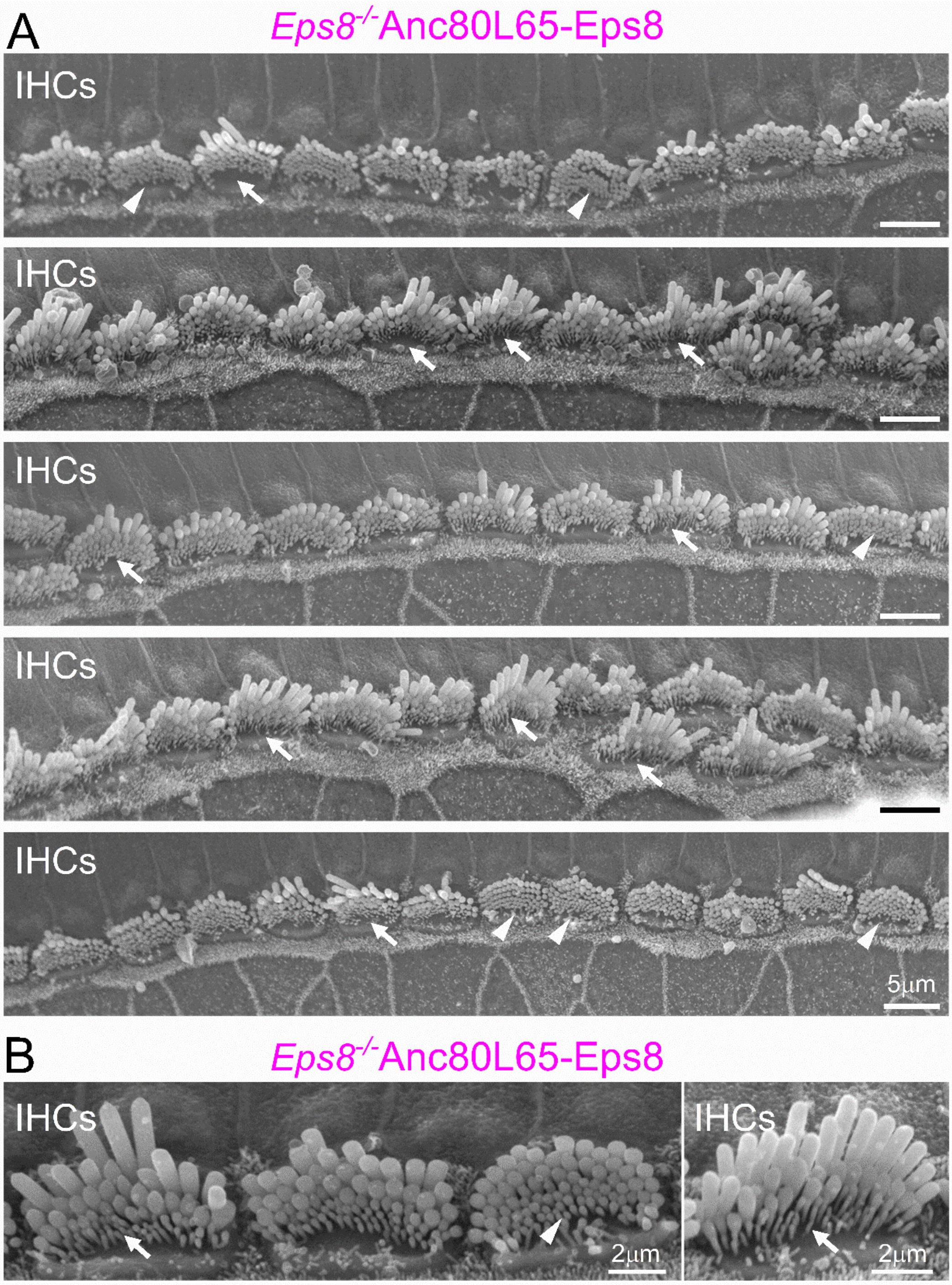

Figure S1. Hair bundle morphology in IHCs from *Eps8*$^{-/-}$ mice transduced with exogenous Eps8. (A) Scanning electron micrographs (SEM) showing examples of the stereociliary bundles of apical-coil IHCs in 5 adult *Eps8*$^{-/-}$ mice that were transduced with Anc80L65-Eps8 *in vivo* at P1-P2. (B) SEM images of the IHC hair bundles at a higher magnification from one the images in Figure 2C (second from the bottom). Arrowheads: indicate examples of hair bundles that show very little or no recovery. Arrows: highlight examples of hair bundles that are considered to have recovered their normal staircase structure with 3 rows of stereocilia.

Figure S2

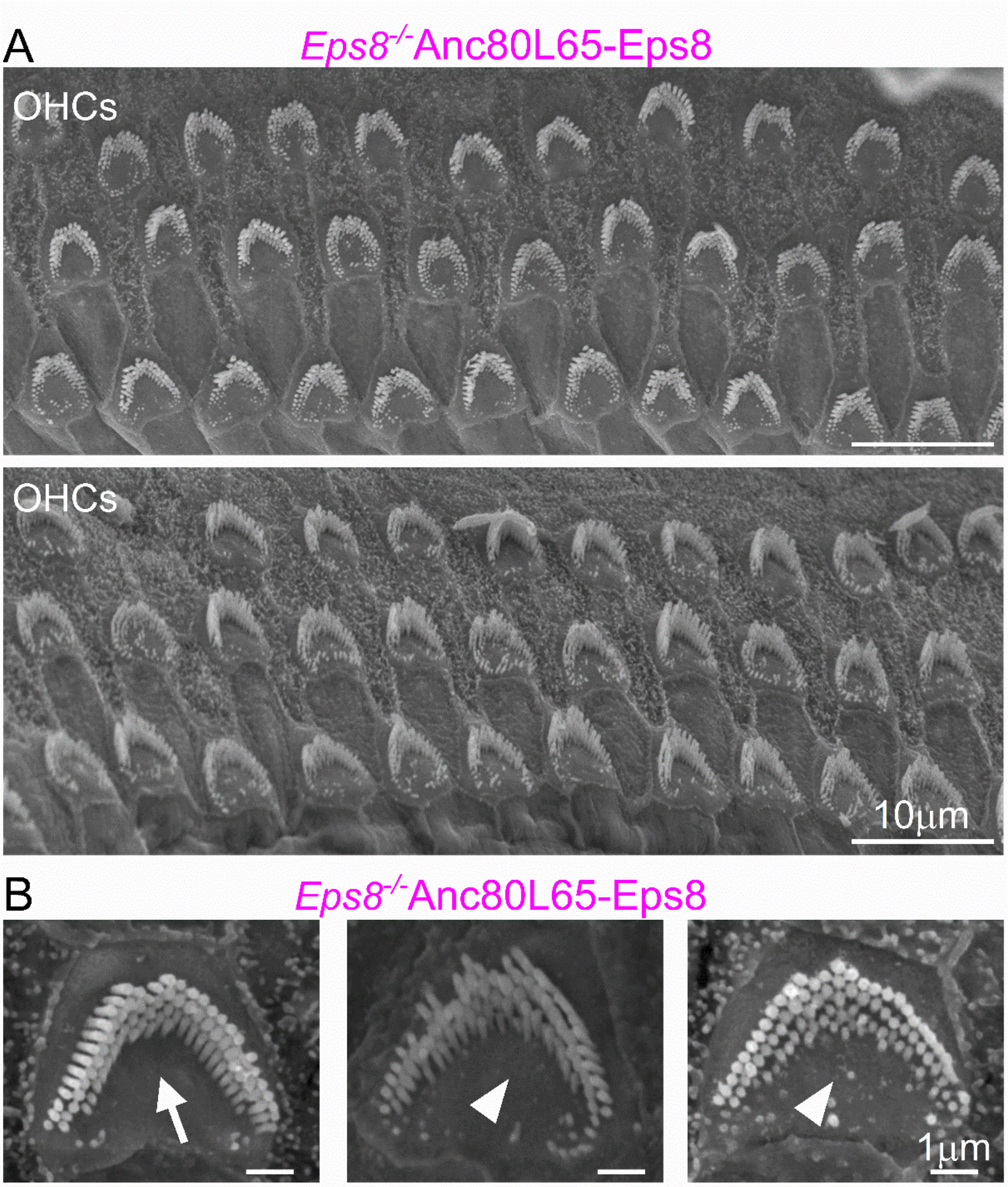

Figure S2. Hair bundle morphology in OHCS from *Eps8*$^{-/-}$ mice transduced with exogenous Eps8.

(A,B) SEM showing examples of the stereociliary bundles of apical-coil IHCs in 2 adult *Eps8*$^{-/-}$ mice that were transduced with Anc80L65-Eps8 *in vivo* at P1-P2. Arrowheads: indicate hair bundles that show very little recovery. Arrow: highlight a hair bundle that was considered to have recovered its normal staircase structure with 3 rows of stereocilia.

Figure S3

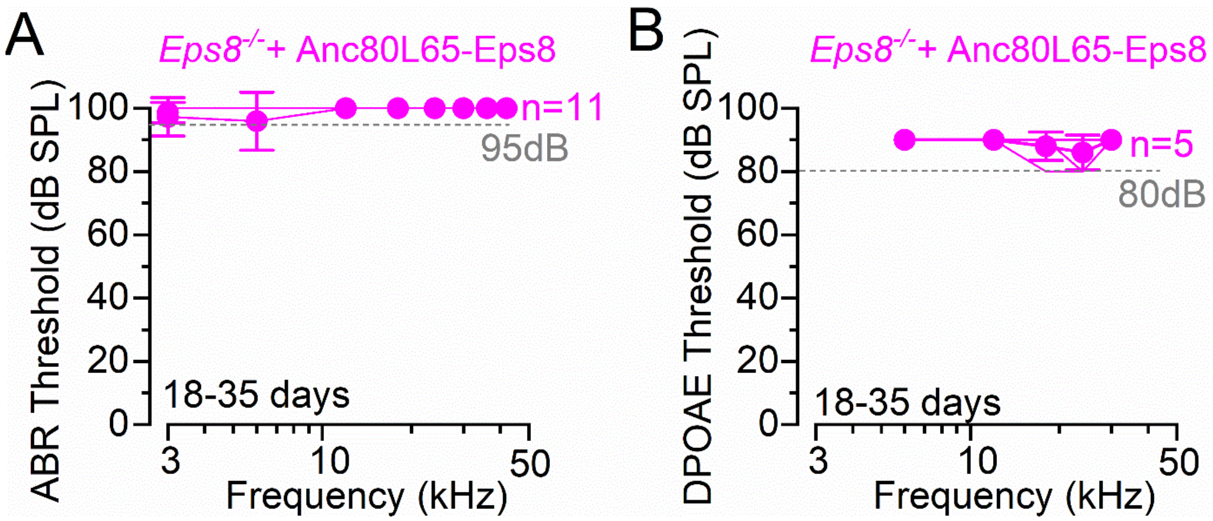

Figure S3: Anc80L65-Eps8 do not rescue hearing in *Eps8*$^{-/-}$ mice.

(A) ABR thresholds for frequency-specific pure tone stimulation from 3 kHz to 42 kHz recorded from *Eps8*$^{-/-}$ mice injected at P1-P2 with Anc80L65-Eps8 (P21-P35). The number of mice tested is shown. The dashed line represents the upper threshold limit of our system, 95 dB. Single animal recordings are plotted as faded lines. (C*)* DPOAE thresholds measured from a subset or all the mice also used in panel (A). The frequency range tested was between 6 kHz and 24 kHz. Dashed line: upper threshold limit of the system, 80 dB. Single animal recordings are shown as faded lines. Data are plotted as means ± SD.

Figure S4

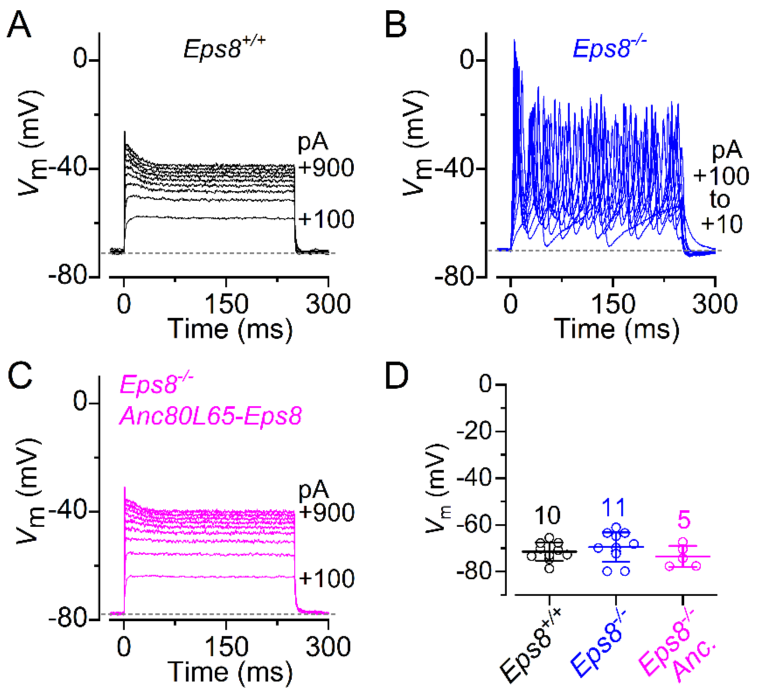

Figure S4. Rescue of IHC voltage responses in *Eps8*$^{-/-}$ mice injected with Anc80L65-Eps8

(A-C) IHC voltage responses elicited by applying a series of depolarizing current injections (10 pA or 100 pA increments), from their respective membrane potentials, from *Eps8*$^{+/+}$ (P19: A), *Eps8*$^{-/-}$ (P20: B) and *Eps8*$^{-/-}$ injected with Anc80L65-Eps8 (P22, C) mice. Note that while *Eps8*$^{+/+}$ and rescued *Eps8*$^{-/-}$ mice exhibited normal graded and sustained responses to current injections, *Eps8*$^{-/-}$ retained the characteristic spiking behaviour that is normally only present during in pre-hearing stages. (D) Average IHC resting membrane potential (V_m) was not significantly different between the different mouse models (P = 0.3487, one-way ANOVA). Single-cell V_m values (open symbols) are plotted behind the average data. Number of IHCs investigated is shown above the average data points. Data are plotted as means ± SD.

Figure S5

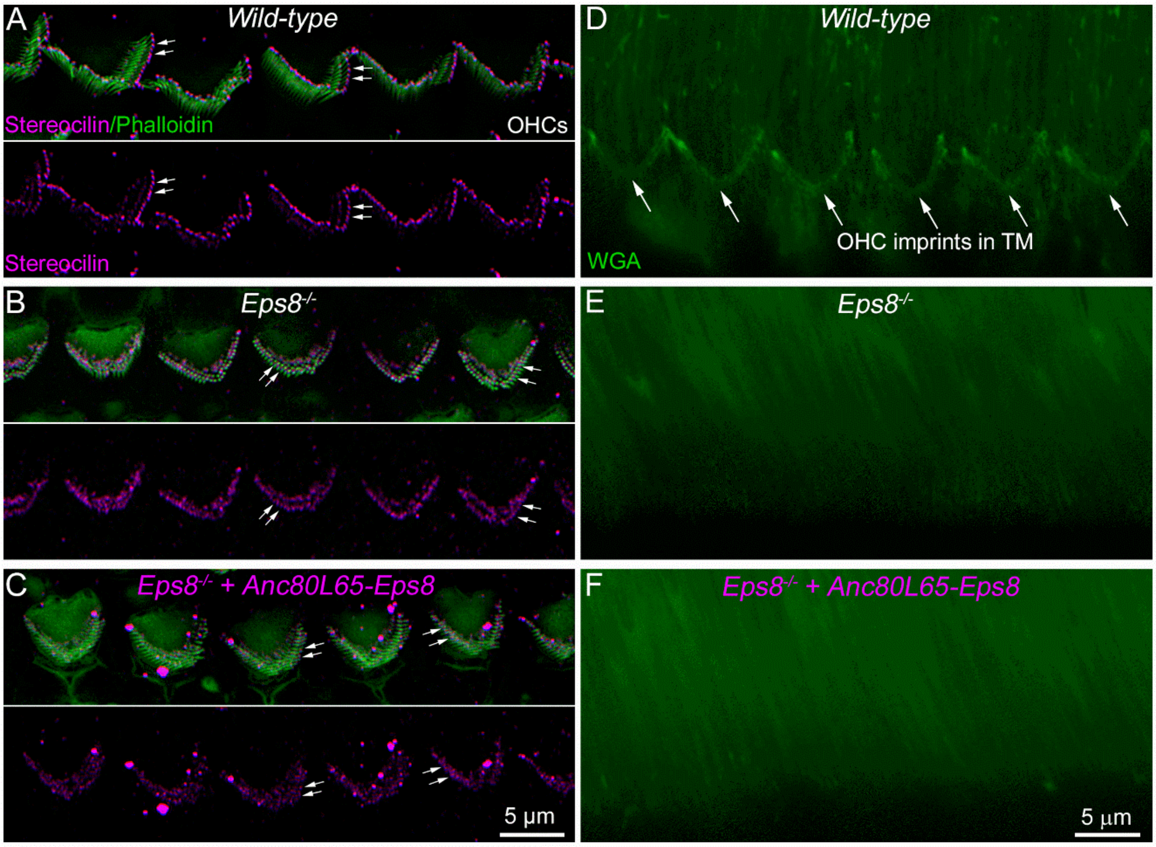

Figure S5. OHC stereocilia from rescued *Eps8*$^{-/-}$ mice fail to attach to the tectorial membrane (A-C**)** OHCs from whole-mount preparations of adult wild-type (A), *Eps8*$^{-/-}$ (B) and *Eps8*$^{-/-}$ rescued (C) cochleae double-labelled with an anti-stereocilin antibody (magenta) and phalloidin (green). Images are generated from 0.6 micron Z-projections from super-resolution confocal scans encompassing the upper regions of the stereocilia. Strongest stereocilin label is seen at the tips of the tallest row of stereocilia in the wild-type (A, arrows), whilst in the shortened hair bundles of the *Eps8*$^{-/-}$ stereocilin labelling is only present between the stereocilia (B, arrows) and not at the tips of the stereocilia that are expected to adhere to tectorial membrane (TM). In the *Eps8*$^{-/-}$ mice rescued with Anc80L65-Eps8, although hair bundles are observed with the normal staircase pattern of stereocilia rows, stereocilin is absent from the tips of the tallest row and is only observed between the stereocilia (C, arrows). (D-F) Airyscan confocal Z-projections (1.4 microns in depth) from mid-coil regions of the TM stained with FITC-conjugated wheat-germ agglutinin. Each projection is from the underside of the TM, in the region where the imprints from OHCs of row 1 would be present. Clear imprints are readily visible in the wild-type (arrows) but none are observed in the *Eps8*$^{-/-}$ (E) or *Eps8*$^{-/-}$ Anc80L65-Eps8 rescued (F) cochlea.

Figure S6

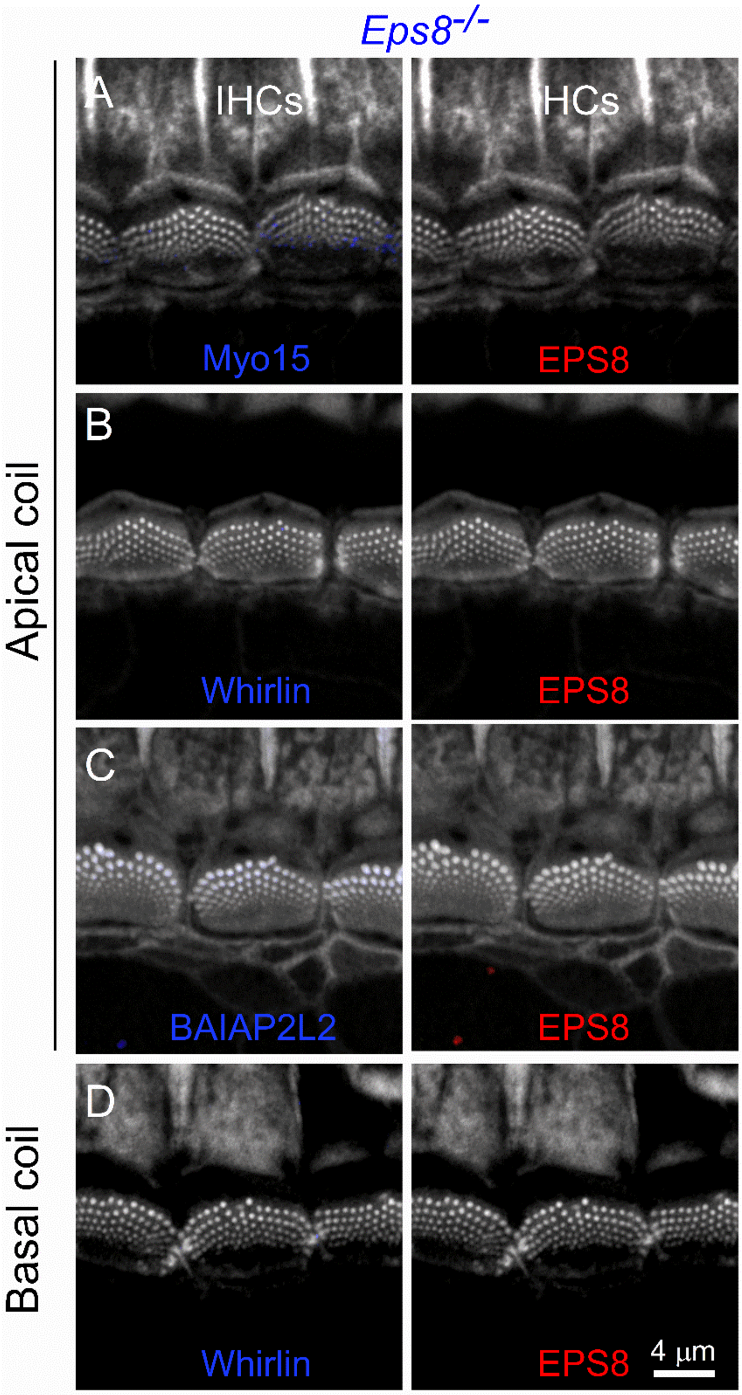

Figure S6. Stereocilia from *Eps8*$^{-/-}$ mice do not express the characteristic bundle proteins
(A-D) Confocal images of the hair bundles from apical-coil (A-C) and basal-coil (D) IHCs of >P20 *Eps8*$^{-/-}$ mice immunostained for EPS8 (red) together with MYO15 (A), WHIRLIN (B,D) and BAIAPL2 (C) (blue). Stereocilia are labelled with phalloidin (white). None of the above proteins is expressed at the stereocilia.
